# Clinician-Driven Reanalysis of Exome Sequencing Data From Patients With Inherited Retinal Diseases

**DOI:** 10.1001/jamanetworkopen.2024.14198

**Published:** 2024-05-31

**Authors:** Dongheon Surl, Dongju Won, Seung-Tae Lee, Christopher Seungkyu Lee, Junwon Lee, Hyun Taek Lim, Seung Ah Chung, Won Kyung Song, Min Kim, Sung Soo Kim, Saeam Shin, Jong Rak Choi, Riccardo Sangermano, Suk Ho Byeon, Kinga M. Bujakowska, Jinu Han

**Affiliations:** 1 Institute of Vision Research, Department of Ophthalmology, Gangnam Severance Hospital, Yonsei University College of Medicine, Seoul, South Korea; 2 Department of Laboratory Medicine, Severance Hospital, Yonsei University College of Medicine, Seoul, South Korea; 3 Institute of Vision Research, Severance Hospital, Department of Ophthalmology, Yonsei University College of Medicine, Seoul, South Korea; 4 Seoul Orthopia Eye Clinic, Seoul, South Korea; 5 Department of Ophthalmology, Ajou University School of Medicine, Suwon, South Korea; 6 Gangnam Yonsei Eye Clinic, Seoul, South Korea; 7 Ocular Genomics Institute, Massachusetts Eye and Ear Infirmary, Department of Ophthalmology, Harvard Medical School, Boston

## Abstract

**Importance:**

Despite advances in next-generation sequencing (NGS), a significant proportion of patients with inherited retinal disease (IRD) remain undiagnosed after initial genetic testing. Exome sequencing (ES) reanalysis in the clinical setting has been suggested as one method for improving diagnosis of IRD.

**Objective:**

To investigate the association of clinician-led reanalysis of ES data, which incorporates updated clinical information and comprehensive bioinformatic analysis, with the diagnostic yield in a cohort of patients with IRDs in Korea.

**Design, Setting, and Participants:**

This was a multicenter prospective cohort study involving 264 unrelated patients with IRDs, conducted in Korea between March 2018 and February 2020. Comprehensive ophthalmologic examinations and ES analyses were performed, and ES data were reanalyzed by an IRD specialist for single nucleotide variants, copy number variants, mobile element insertions, and mitochondrial variants. Data were analyzed from March to July 2023.

**Main Outcomes and Measures:**

Diagnostic rate of conventional bioinformatic analysis and clinician-driven ES reanalysis.

**Results:**

A total of 264 participants (151 [57.2%] male; mean [SD] age at genetic testing, 33.6 [18.9] years) were enrolled, including 129 patients (48.9%) with retinitis pigmentosa and 26 patients (9.8%) with Stargardt disease or macular dystrophy. Initial bioinformatic analysis diagnosed 166 patients (62.9%). Clinician-driven reanalysis identified the molecular cause of diseases in an additional 22 patients, corresponding to an 8.3–percentage point increase in diagnostic rate. Key factors associated with new molecular diagnoses included clinical phenotype updates (4 patients) and detection of previously overlooked variation, such as structural variants (9 patients), mitochondrial variants (3 patients), filtered or not captured variants (4 patients), and noncanonical splicing variants (2 patients). Among the 22 patients, variants in 7 patients (31.8%) were observed in the initial analysis but not reported to patients, while those in the remaining 15 patients (68.2%) were newly detected by the ES reanalysis.

**Conclusions and Relevance:**

In this cohort study, clinician-centered reanalysis of ES data was associated with improved molecular diagnostic yields in patients with IRD. This approach is important for uncovering missed genetic causes of retinal disease.

## Key Points

**Question:**

Is clinician-led reanalysis of exome sequencing data, incorporating clinical information and bioinformatic analysis, associated with improved diagnosis for patients with inherited retinal disease (IRD) without a molecular diagnosis?

**Findings:**

In this cohort study involving 264 unrelated patients with IRDs in Korea, clinician-driven reanalysis identified the molecular cause of diseases in an additional 22 patients, corresponding to an 8.3–percentage point increase in diagnostic rate. Contributing factors associated with new molecular diagnoses included updates of clinical phenotype, structural variants, mitochondrial variants, filtered or not captured variants, and noncanonical splicing variants.

**Meaning:**

These findings suggest that clinician-centered reanalysis of exome sequencing data was associated with improved molecular diagnostic yields in patients with IRDs.

## Teaser

This cohort study examines the association of clinician-driven exome sequencing data reanalysis, which incorporates updated clinical information and comprehensive bioinformatic analysis, with diagnostic yield in patients with inherited retinal disease in South Korea.

## Introduction

Inherited retinal diseases (IRDs) are a group of clinically and genetically heterogeneous diseases characterized by progressive loss of vision and visual fields caused by dysfunction of the retina.^1^ Approximately 1 in 2000 individuals, or more than 2 million people worldwide, are affected by IRDs,^2^ and the phenotypes vary, ranging from blindness to mild impairment of visual acuity. To date, more than 280 genes have been identified for IRD, and clinical application of next-generation sequencing (NGS) plays a key role in genetic diagnostics and new IRD gene discovery.^1,3^ Despite these technical advancements, 30% to 50% of individuals with IRD do not receive definite molecular diagnosis after genetic testing.^4^ There are several reasons to explain missing heritability in IRDs, including deep intronic variants, noncoding regulatory changes, variants in repetitive low-complexity sequence regions, mobile element insertions (MEIs), copy number variations (CNVs), or complex structural variants, all of which can be identified through genome sequencing (GS). However, the diagnostic increase associated with GS compared with exome sequencing (ES) is still minor.^5,6^ A 2024 study suggested that GS diagnoses were made in 28% of ES-unresolved families, but repeated ES with a contemporary pipeline identified most small nucleotide variants and CNVs undetected in older ES.^7^ Moreover, the massive amount of data generated from GS is fastidious to analyze without sophisticated computational tools. Therefore, narrow pretest hypothesis and sequence data reanalysis by well-trained clinicians or clinical geneticists are imperative for specific molecular diagnosis even after GS becomes a mainstay diagnostic tool.

It has been argued that ES reanalysis should be performed routinely in the clinical setting, as it has the potential to yield additional diagnoses. This potential primarily arises from newly discovered gene-disease relationships, updated clinical information, and advancement of bioinformatics tools.^8,9,10,11^ In most clinics specializing in IRDs, bioinformatic analysis, variant curation process, and delineation of the retinal phenotype are usually carried out independently. While effective communication among clinicians, variant curators, and bioinformaticians is crucial for accurate molecular diagnosis, sustaining ongoing communication within the team is often impractical due to time constraints. Thus, we hypothesized that a holistic, clinician-driven whole analysis, including bioinformatic reanalysis, variant annotation, and genotype-phenotype matching, could increase diagnostic yield. We conducted a prospective study on the clinical utility of ES reanalysis in a consecutive series of 264 patients with IRD, comparing the diagnostic yield of a conventional approach with that of ophthalmologist-led comprehensive ES reanalysis. We also present a genotype-phenotype spectrum of IRDs observed in a South Korean cohort.

## Methods

This cohort study was approved by the institutional review board of Severance Hospital, Yonsei University College of Medicine, and adhered to the tenets set forth in the Declaration of Helsinki. All participants provided written informed consent. This study followed the Strengthening the Reporting of Observational Studies in Epidemiology (STROBE) reporting guideline.

### Patient Recruitment and Phenotypic Data Collection

This multicenter prospective consecutive study included 264 unrelated patients with IRDs who underwent ES between March 2018 and February 2020 in Korea. Patients underwent ophthalmologic examinations, including slitlamp examination, fundus examination, autofluorescence examination, determination of the presence and type of nystagmus, and determination of presence of other systemic symptoms. Optical coherence tomography (Heidelberg Engineering), and electroretinography (RETI-port, Roland Consult, or RetEval, LKC Technologies) were performed. Peripheral blood samples were isolated from patients for genetic analysis. Whole blood was collected in ethylenediaminetetraacetic acid tubes, and genomic DNA was extracted within 24 hours at room temperature. QIAamp DNA Blood Mini Kit (Qiagen) was used for DNA isolation in accordance with the manufacturer’s instruction. Race and ethnicity were assessed by investigator-observation, and all participants were Korean. Race and ethnicity were assessed because collection of data on race and ethnicity was required by the funding agency.

### Sequencing and Bioinformatic Analysis

ES was performed on a NovaSeq 6000 system (Illumina) at a core facility (DNA Link) using an IDT xGen Exome Research Panel V1 (145 patients), Twist Human Core Exome (118 patients), or Agilent SureSelect V6 (1 patient). The median (IQR) depth of coverage was 117.6× (IQR, 84.1× to 162.5×). The sequencing data analysis was conducted by 2 separate investigators, the bioinformatic analysis team (D.W. and S.T.L.) and an experienced pediatric ophthalmologist (J.H.). Two investigators used different bioinformatic algorithms; detailed description of initial bioinformatic analysis were described previously.^12,13^ Briefly, the demultiplexed fastq files were aligned with hg19 reference genome using the Burrows-Wheeler Aligner programs, followed by removal of duplicate reads, and base quality recalibration, and Haplotypecaller was conducted using the Genome Analysis ToolKit (GATK)version 3.8 (Broad Institute). The Variant Call Format (VCF) files were annotated with Variant Effect Predictor (Ensembl) and ANNOVAR (ANNOVAR) software.^14^ Each variant suspected to be pathogenic, likely pathogenic, or a variant of uncertain significance (VUS) was confirmed with visual inspection of the bam file using Integrative Genomics Viewer software version 2.16 (University of California, San Diego, and the Broad Institute). Split-read–based detection of large structural variation was conducted using Pindel.^15^ Read-depth based detection of CNVs was conducted using ExomeDepth R package version 1.1.10 (R Project for Statistical Computing), followed by visualization using a base-level read depth normalization algorithm.^16,17^ Copywrite R version 2.9.0 was used with a 1-megabase (Mb) window option for off-target analysis and whole chromosomal CNV detection.^18^

### ES Reanalysis and Resequencing

The raw data for all patients were reanalyzed by a single clinician (J.H.) annually. Final reanalysis was conducted in March to July 2023, 3 to 5 years after the initial analysis. The demultiplexed fastq files were aligned with alt-masked hg38 reference genome using dragen mapper version 1.3.0 (Illumina). Then, mark duplication and Haplotypecaller with DRAGEN-GATK best practice were conducted using the GATK version 4.4.0.0. The genomic VCF files were imported into genomicsDBimport (GATK), and joint calling was performed. The joint calling VCF file was uploaded into Seqr (Broad Institute) and subsequently annotated with Variant Effect Predictor.^14^ MEI analysis was done with SCRAMble (GeneDx).^19^ A customized grep program developed by our group was also applied to detect Alu insertion in *RP1*, a common insertion in East Asian.^20^ Read-depth based detection of CNVs was conducted using R packages cn.Mops, ExomeDepth version 1.1.16, and GATK gCNV (eAppendix 1y, eFigure 1, and eFigure 2 in Supplement 1).^17,21,22^

Additional long-range PCR Sanger sequencing in *RPGR* ORF15 regions was conducted in 17 patients with early onset severe retinal dystrophy or suspected X-linked retinitis pigmentosa (RP) without known genetic explanation. Familial cosegregation analysis using Sanger sequencing or multiplex ligation-dependent probe assay (MLPA) was performed if feasible. Minigene splicing assays were conducted to confirm aberrant splicing (eAppendix 2-4 and eFigure 3 in Supplement 1).

### Variant Filtering and Classification

A minor allele frequency (MAF) in the Genome Aggregation Database (gnomAD; version 2.1.1 for initial analysis and version 3.1.2 for ES reanalysis) for each variant was determined. The variants in known IRD genes were analyzed (eAppendix 5 in Supplement 1). An MAF of more than 5% in autosomal recessive disease and more than 0.02% in autosomal dominant disease were excluded (eAppendix 6 and eFigure 1 in Supplement 1). The pathogenicity of variants was estimated using 4 in silico algorithms: SIFT, PolyPhen-2, combined annotation dependent depletion, and functional analysis through hidden markov models.^23,24,25,26^ Variants within splice sites and deep intronic regions were evaluated using Alamut splicing module and SpliceAI (Illumina).^27^ The pathogenicity of single nucleotide variations, small indels, and CNVs were determined in accordance with the 2015 guidelines of the American College of Medical Genetics and Association for Molecular Pathology 2015 and 2020 joint consensus of the American College of Medical Genetics and Clinical Genome Resource.^28,29^ Automated classification was performed using Franklin by Genoox,^30^ and manual adjustment was done when there were conflicting results (eFigure 4 in Supplement 1).

Molecular diagnosis was defined on the basis of the inheritance pattern, zygosity, pathogenicity of the variant, and genotype-phenotype correlation. Variants were deemed diagnostic if they were pathogenic, likely pathogenic in a gene associated with the patient’s phenotype, or VUS in a gene associated with the phenotype but with unavailable parental segregation data.

## Results

### Patient Demographics

Among 264 included patients with IRD, all were of Korean ethnicity, 152 (57.6%) were male, and none of the patients were of consanguineous parentage. The mean (SD) age at genetic testing was 33.6 (18.9) years, with a range of 0.7 to 89.7 years and a median (IQR) age of 32.7 (19.0 to 48.9) years) (eFigure 5 in Supplement 1). There were 91 patients (34.5%) with a family history of IRDs, and the remaining 173 patients (65.5%) did not have a family history of IRD. Patients were phenotypically heterogeneous: nystagmus was present in 63 patients (23.9%), and systemic features, including hearing loss, were found in 34 patients (12.9%) (eTable 1 in Supplement 1).

### Diagnostic Performance of ES Reanalysis and Variation Spectrum

**Figure 1.**
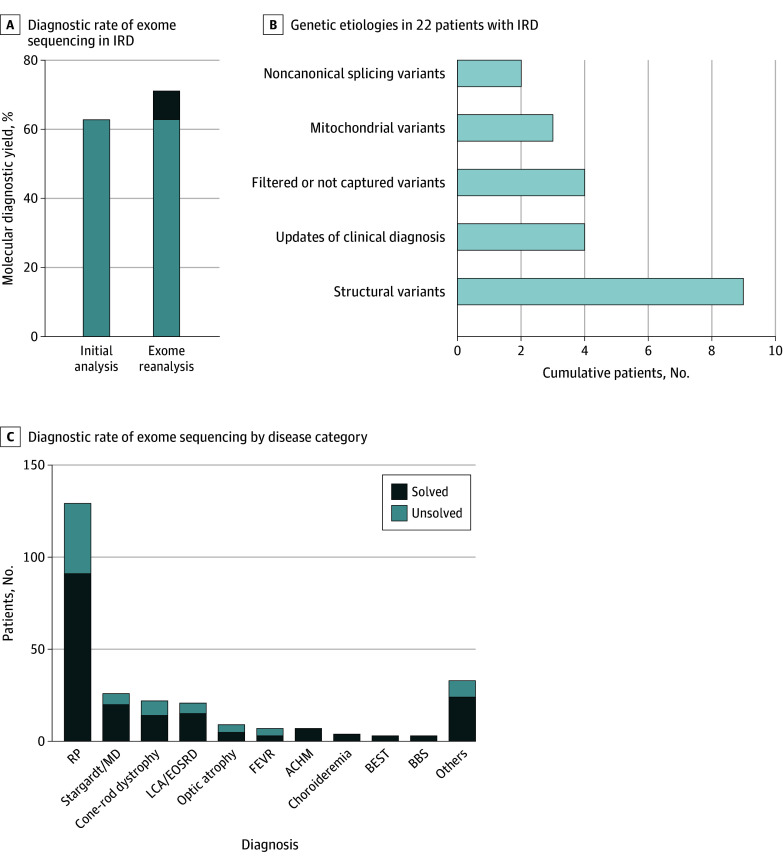
The Diagnostic Rate, Genotypes, and Phenotypes of the Cohort A, Diagnostic rate of exome sequencing in patients with inherited retinal diseases (IRD). B, Exome sequencing reanalysis identified genetic etiologies in 22 patients with IRD. The additional diagnostic uplift was 8.3 percentage points. C, Diagnostic rate of exome sequencing in each disease category. ACHM indicates achromatopsia; BBS, Bardet-Biedl syndrome; BEST, bestrophinopathy; EOSRD, early onset severe retinal dystrophy; FEVR, familial exudative vitreoretinopathy; LCA, Leber congenital amaurosis; MD, macular dystrophy; RP, retinitis pigmentosa.

Initial bioinformatic analysis resulted in molecular diagnoses for 166 patients (62.9%), with the remaining 98 patients with no molecular diagnosis. The clinician-driven bioinformatic reanalysis was associated with further molecular diagnoses for 22 patients (8.3%) (Figure 1 and Table). Consequently, overall diagnostic rate of ES in the studied IRD cohort reached 71.2% (188 of 264 patients). The age at onset of IRD varied among patients with molecular diagnoses, ranging from 0.6 to 45.5 years (eFigure 6 in Supplement 1). The segregation information are shown in eTable 2 and eFigure 7 in Supplement 1. In total, 231 unique disease-associated genetic variants were discovered from 188 probands with molecular diagnosis (eTable 2 in Supplement 1). Of these, 66 variants (28.6%) were novel (eTable 3 in Supplement 1). Collectively, 65 IRD-associated genes were responsible for variations in this cohort. Disease-associated variants were most frequently found in *EYS* (23 variants), followed by *ABCA4* (22 variants) and *USH2A* (19 variants) (eFigure 8 in Supplement 1). The results of in silico analyses of splicing variants are described in eTable 4 in Supplement 1.

**Table.** The Results of Clinician-Driven ES Reanalysis

Finding by patient identification No.	Sex	Age, y	Diagnosis	Gene	Causative variants	CADD	gnomAD^a^	Reason for the negative ES findings
Update of clinical diagnoses								
SB_0032	F	30s	RP	*IMPG1*	c.1896T>G:p.(Ser632Arg)	23.7	Not found	Phenotypic expansion
GH_0182	F	<20	Optic atrophy	*PTPN23*	c.3768del:p.(Pro1258Argfs*2)^b^^,^^c^; c.4886C>G:p.(Pro1629Arg)^b^^,^^c^	31; 25.5	Not found; not found	Not listed in IRD genes
SH_0059	M	30s	Optic atrophy and cone dystrophy	*NBAS*	c.3494del:p.(Val1165Serfs*31)^b^^,^^c^; c.5740C>T:p.(Arg1914Cys)^b^^,^^c^	34; 24.9	Not found; not found	Not listed in IRD genes
GH_0198	M	50s	FEVR	*TSPAN12*	c.194C>T:p.(Pro65Leu)	22.9	2/251238	Revised clinical diagnosis
Structural variants								
GS_0083	F	20s	RP	*USH2A*	c.2802T>G:p.(Cys934Trp); exon 47 deletion^b^	25.5; NA	57/282482; Not found	Missed single exon CNV
GH_0044	F	<10	LCA	*NMNAT1*	c.709C>T:p.(Arg237Cys); exon 2 deletion^b^^,^^c^	35; NA	14/277114; Not found	Missed single exon CNV
GJ_0161	M	40s	RP	*ARSG*	c.982+1G>C; exon 5 deletion^b^	25.8; NA	1/250984; Not found	Missed single exon CNV
SS_0039	M	20s	Choroideremia	*CHM*	Exon 2-8 duplication^b^^,^^c^	NA	Not found	Not reported
SH_0066	M	40s	Macular dystrophy	*RP1*	c.4052_4053ins328^d^; c.5797C>T:p.(Arg1933*)	NA; 38	Not found; 49/281934	Mobile element insertions
GS_0037	F	20s	Macular dystrophy	*RP1*	c.4052_4053ins328^d^; c.5797C>T:p.(Arg1933*)	NA; 38	Not found; 49/281934	
GH_0072	M	<20	Cone rod dystrophy	*RP1*	c.4052_4053ins328^d^; c.4582_4585del:p.(Ile1528Valfs*10)	NA; 25.7	Not found; 4/281542	
GH_0021	M	<20	Cone rod dystrophy	*RP1*	c.4052_4053ins328^d^; c.4196del:p.(Cys1399Leufs*5)	NA; 22.1	Not found; 1/250622	
GS_0191	F	40s	Macular dystrophy	*RP1*	c.4052_4053ins328^d^; c.5797C>T:p.(Arg1933*)	NA; 38	Not found; 49/281934	
Mitochondrial variants								
GH_0146	M	30s	RP	*MT-ATP6*	m.8993T>G (72% heteroplasmy)	24	Not found	Mitochondrial variants
GH_0184	F	30s	RP	*MT-ATP6*	m.8993T>G (74% heteroplasmy)	24	Not found	
GH_0201	M	30s	Optic atrophy	*MT-ND4*	m.11778G>A (homoplasmy)	24.4	11/56423	
Filtered or not captured variants								
SH_0008	M	<20	Ocular albinism	*GPR143*	c.703G>A:p.(Glu235Lys)	27.7	Not found	Filtered variant
GH_0131	M	30s	LCA	*PROM1*	c.1192C>T:p.(Gln398*)^b^; c.1877_1878del:p.(Ile626Argfs*6)	31; 32	Not found; 1/249108	Not captured variant
GH_0077	M	<20	RP	*RPGR*	c.2937_2938del:p.(Glu980Glyfs*98)	24	Not found	Filtered variant
GH_0068	M	30s	RP	*RPGR*	c.2961_2968dup:p.(Gly990Glufs*102)^b^	23.5	Not found	Not called variant
Noncanonical splice site variants								
GH_0054	F	<20	Cone dystrophy	*RAB28*	c.68C>T:p.(Ser23Phe); c.76-158T>G^b^	26; 12.13	7/219238; Not found	Deep intronic variant detected in ES
SH_0058	F	30s	RP	*CNGB1*	c.217+5G>C; c.2154C>T:p.(Gly718=)	23.2; 15.9	13/280826; 12/248428	Synonymous splicing variant

Abbreviations: CADD, combined annotation dependent depletion; CNV, copy number variant; ES, Exome sequencing; F, female; FEVR, familial exudative vitreoretinopathy; gnomAD, Genome Aggregation Database; IRD, inherited retinal disease; LCA, Leber congenital amaurosis; M, male; NA, not available; RP, retinitis pigmentosa.

^a^ The gnomAD version 2.1.1. was used to check minor allele frequency of the autosomal variants, and gnomAD version 3.1 was used to check minor allele frequency of mitochondrial variation.

^b^ These variants were novel.

^c^ These variants were novel but previously reported by our group.

^d^ c.4052_4053ins328:p.(Tyr1352Alafs*9) *Alu* element insertion in *RP1* exon 4.

### Factors Associated With New Molecular Diagnoses

ES reanalysis was associated with new molecular diagnoses in 22 patients in this cohort. Important factors associated with these new molecular diagnoses included a thorough clinical examination leading to updated patient diagnoses and phenotype expansion and detection of previously overlooked structural variants, including mobile element insertions, mitochondrial variants, filtered or not captured variants in ES, and deep intronic or synonymous variants causing missplicing (Table and Figure 1B). Among 22 patients with new molecular diagnoses, the causative variants in 7 patients (31.8%) were observed in the initial bioinformatic analysis and became diagnostic afterwards. The causative variants in the remaining 15 patients (68.2%) were newly discovered using the updated bioinformatic pipelines.

### Update of Clinical Diagnoses

Four patients received new molecular diagnosis through a clinical reanalysis prompted by genetic findings. In a patient with sectoral RP (patient identifier: SB_0032), a single heterozygous c.1896T>G:p.(Ser632Arg) variant in *IMPG1* was detected. While *IMPG1* variations are traditionally linked to a dominant form of vitelliform macular dystrophy,^31^ a 2021 study found that *IMPG1* variants also were associated with autosomal dominant RP.^32^ This insight led to the reclassification of the identified variant as a candidate for causing disease. Notably, this variant is not present in gnomAD and TopMed, and the altered amino acid at position 632 is located at the sperm protein, enterokinase, and agrin domain, which is highly conserved across species, from zebrafish to human. Several in silico analysis tools indicate that this variant is likely deleterious (eTable 3 in Supplement 1), leading us to label this variant as diagnostic (eFigure 9 in Supplement 1).^31,32^ In another patient with RP (patient identifier: GH_0198), a heterozygous c.194C>T:p.(Pro65Leu) variant in *TSPAN12* was initially deemed nondiagnostic due to a discrepancy between the phenotype and the known genotype associations. Variations in *TSPAN12* are known to be associated with familial exudative vitreoretinopathy (FEVR).^33^ Indeed, a reevaluation of the clinical data revealed temporal retinal vessel dragging in the patient, a hallmark of FEVR, prompting a revised clinical diagnosis to FEVR. Since the c.194C>T:p.(Pro65Leu) variant had previously been classified as likely pathogenic,^34,35^ this allowed us to offer a definite genetic diagnosis to the patient (eFigure 9 in Supplement 1). Details of 2 additional patients with *NBAS* and *PTPN23* variants have been published previously.^36,37^

### Structural Variants

**Figure 2.**
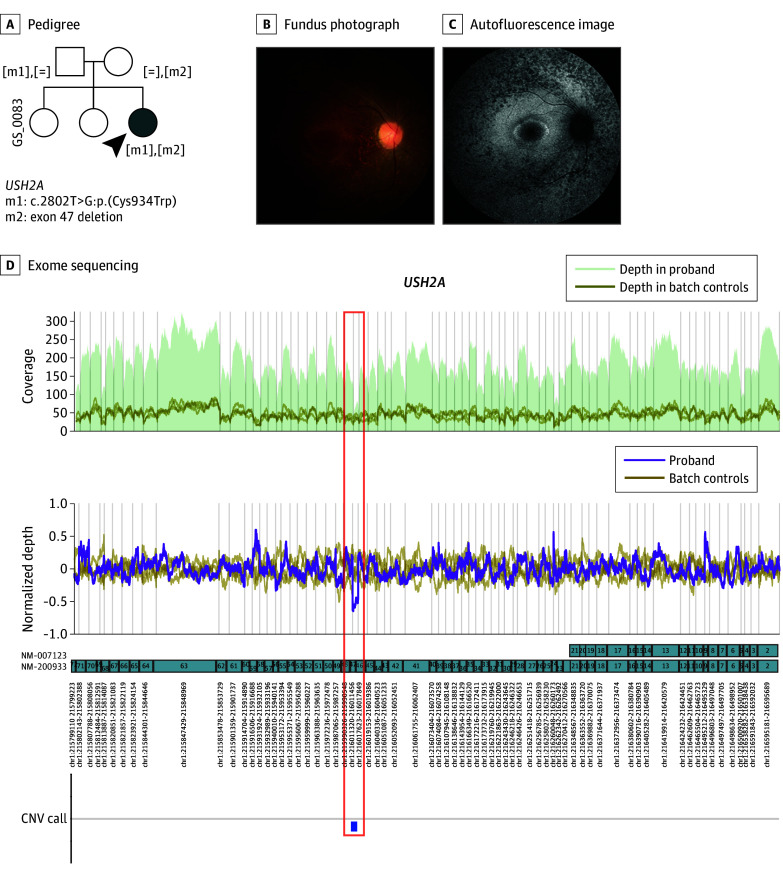
Exome Sequencing Reanalysis Discovered Hidden Structural Variants A, A patient with retinitis pigmentosa with compound heterozygous variants in *USH2A*. Pedigree shows autosomal recessive inheritance pattern. Fundus photograph (B) shows retinal dystrophy, and autofluorescence image (C) shows multiple hypoautofluorescence dots in midperiphery. D, Exome sequencing reanalysis identified exon 47 deletion in *USH2A* (orange box).

ES reanalysis uncovered new disease-causing structural variants in 9 patients (eFigure 10 in Supplement 1). Collectively, the initial analysis and subsequent ES reanalysis identified disease-associated structural variants, including MEIs, in 22 individuals. The American College of Medical Genetics and Clinical Genome Resource scoring of CNVs is summarized in eTable 5 in Supplement 1. For instance, a heterozygous c.2802T>G variant in *USH2A* was found in a patient with RP (patient identifier: GS_0083) during the initial analysis, with no second variant identified. However, ES reanalysis revealed exon 47 deletion in *USH2A*. This single exon deletion was also detected in the unaffected mother using MLPA (Figure 2).

#### Mitochondrial Variants

**Figure 3.**
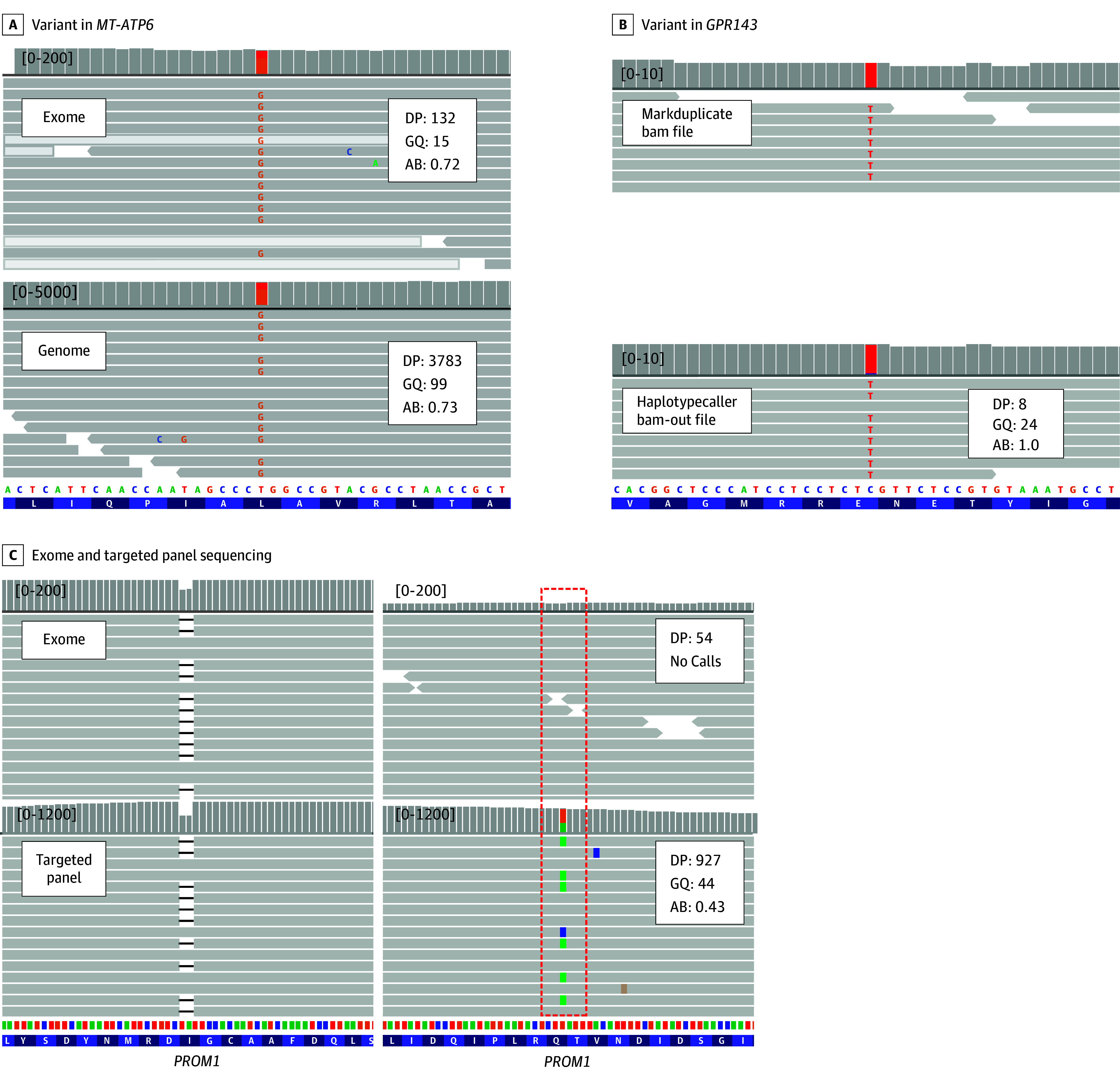
Mitochondrial, Filtered, or Not Captured Variants A, Exome sequencing reanalysis identified mitochondrial variant in a patient with retinitis pigmentosa. The heteroplasmic m.8993T>G variant (72%) in *MT-ATP6* was identified through exome sequencing reanalysis (upper panel). Confirmatory genome sequencing with GATK mitochondrial variant calling best practices also revealed heteroplasmic m.8993T>G (73%) in this patient (lower panel). B, Exome sequencing failed to identify a diagnostic variant at initial analysis, but reanalysis identified c.703G>A:p.(Glu235Lys) variant in *GPR143* (SH_0008). This variant was filtered out due to a depth of 8 in the variant site. C, Integrative Genomic Viewer of BAM files from same patient (upper panel: exome, lower panel: targeted panel sequencing). A man in his 30s (GH_0131), visited our clinic for genetic testing. He presented with nystagmus, and his best corrected visual acuity was hand motion in both eyes. He was clinically diagnosed with Leber congenital amaurosis or early onset severe retinal dystrophy. A study by Ragi et al ^38^ reported that autosomal recessive *PROM1* variants causes Leber congenital amaurosis. However, exome sequencing only identified a single heterozygous frameshift variant in *PROM1* (left panel). Subsequent high-depth targeted panel sequencing was performed, revealing a second c.1192C>T:p.(Gln398*) variant, which was totally missed in exome sequencing data (right panel). AB indicates allelic balance; DP, depth; GQ, genotype quality.

ES reanalysis focusing on mitochondrial variants led to new molecular diagnoses in 3 patients. The heteroplasmic m.8993T>G variant in *MT-ATP6* was detected in 2 patients with RP (patient identifiers: GH_0146 and GH_0184; depth, 132 and 368; heteroplasmy level, 72% and 74%; respectively). In another patient with optic atrophy, the homoplasmic m.11778G>A variant (depth, 31) in *MT-ND4* was identified. A confirmatory test using GS was performed for 1 patient (patient identifier: GH_0146), revealing a heteroplasmic level of 72% in ES, consistent with 73% in GS (Figure 3A). No syndromic features, such as ataxia or neuropathy, were observed in 2 patients carrying a heteroplasmic m.8993T>G variant.

#### Filtered or Not Captured Variants in ES

Through ES reanalysis and targeted resequencing, we identified variants that were filtered out or not captured in 2 patients. In a patient with ocular albinism, a hemizygous c.703G>A:p.(Glu235Lys) variant in *GPR143* was initially filtered due to the low sequence depth of the region (8 reads) (Figure 3B). In another patient with Leber congenital amaurosis or early onset severe retinal dystrophy, ES pinpointed a heterozygous c.1877_1878del:p.(Ile626Argfs*6) variant in *PROM1.* Given that recessive variations in *PROM1* have previously been reported in patients with Leber congenital amaurosis,^38^ and considering the autosomal recessive inheritance pattern observed in this family, we conducted a search for a second variant within the same gene. High-depth targeted panel resequencing unveiled a second c.1192C>T:p.(Gln398*) variant in *PROM1*, which ES had missed despite apparently sufficient coverage of that area (read depth, 54) (Figure 3C).

One male patient with early onset RP (age of onset, 10 years) remained genetically undiagnosed after ES. Considering the early onset of RP, an X-linked RP was suspected. Consequently, long-range PCR Sanger sequencing in *RPGR* ORF15 was used to explore regions inadequately covered by ES. This approach identified a c.2937_2938del:p.(Glu980Glyfs*98) variant in *RPGR* ORF15 (eFigure 11 in Supplement 1). Further examination of 16 patients revealed 1 additional patient with a causative c.2961_2968dup:p.(Gly990Glufs*102) variant (eFigure 11 in Supplement 1). Additionally, 4 other patients were found to carry *RPGR* ORF15 variants that were successfully detected through ES (eTable 2 in Supplement 1).

#### Noncanonical Splicing Variants in ES

**Figure 4.**
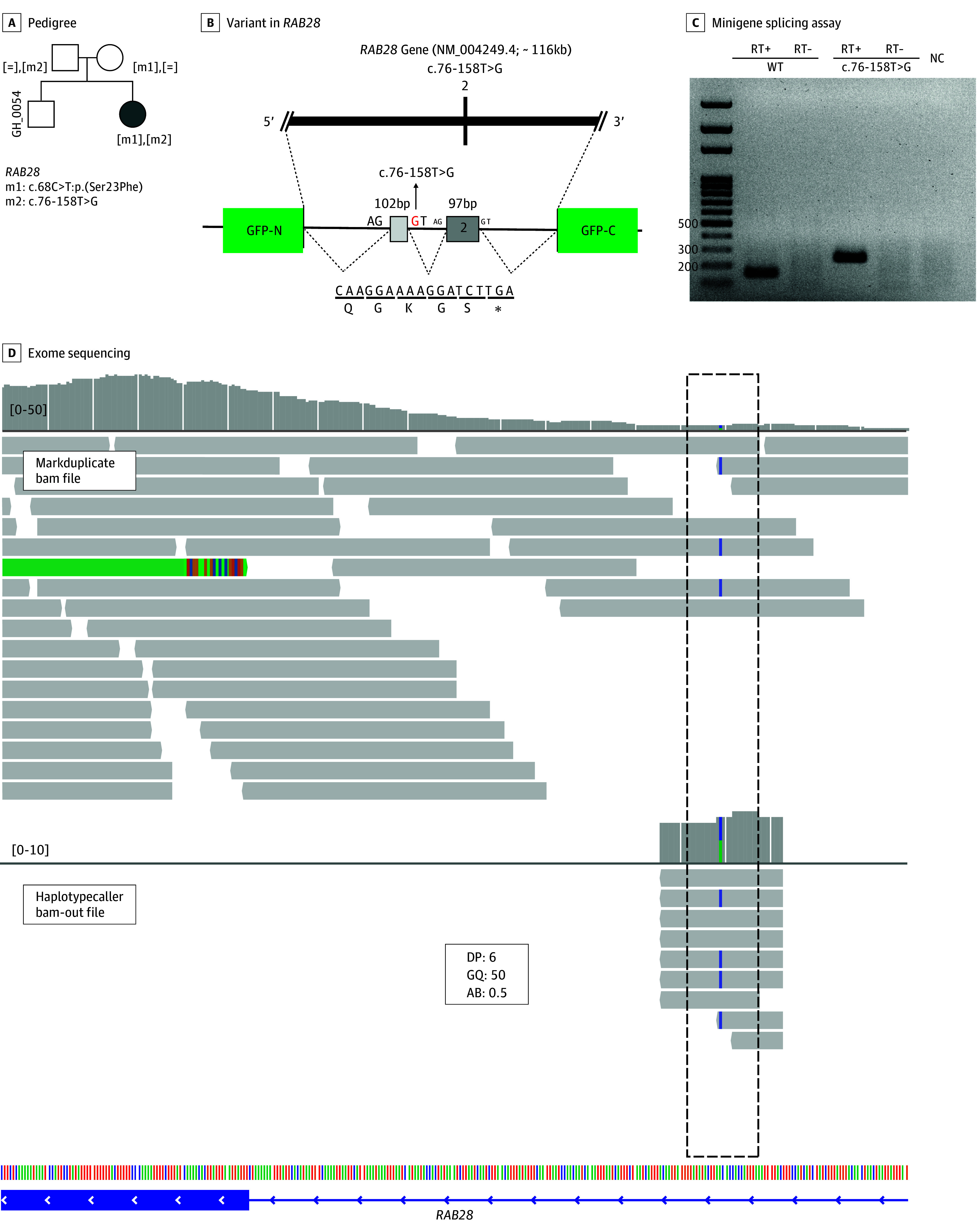
Exome Sequencing Reanalysis Identified Disease-Causing Deep Intronic Variant in a Patient With Cone Dystrophy A, The pedigree showed that proband was the only affected individual in the family. B, Initial exome sequencing revealed a heterozygous c.68C>T:p.(Ser23Phe) variant in *RAB28*. Although depth of the variant was not sufficient (depth, 6), exome sequencing reanalysis showed deep intronic c.76-158T>G variant (SpliceAI: 0.92) in *RAB28*. C and D, Minigene splicing assay confirmed that new cryptic exon (102 bp) was introduced in intron 1, and it may cause premature stop codon. The phase of these 2 variants were confirmed by Sanger sequencing of the parents. AB indicates allelic balance; DP, depth; GQ, genotype quality; NC, negative control.

Initial ES analysis identified a heterozygous c.68C>T:p.(Ser23Phe) variant in *RAB28* in a patient with cone dystrophy. On reanalysis, ES uncovered a rare deep intronic c.76-158T>G variant in *RAB28*, which was captured at a low sequence depth of 6. Segregation analysis with Sanger sequencing confirmed that these 2 variants existed in trans. The c.76-158T>G variant was expected to induce the inclusion of a cryptic exon by analysis with SpliceAI and Alamut splicing module. This expectation was verified by a subsequent minigene splicing assay (Figure 4). In a patient with RP (patient identifier: SH_0058), 2 rare variants were discovered in *CNGB1*. The first was a c.217+5G>C likely pathogenic variant, and the second was a synonymous c.2154C>T:p.(Gly718=) VUS with ClinVar identifier 885333. The synonymous variant was expected to cause 3′ exon truncation by 14 base pairs (bp), introducing a premature stop codon. The expectation was confirmed by the minigene splicing assay (eFigure 12 in Supplement 1).

## Discussion

Our cohort study found that clinician-led ES reanalysis was associated with enhanced molecular diagnostic yields for Mendelian diseases, increasing the diagnostic rate for this IRD cohort from 62.9% to 71.2%. The main factors associated with diagnostic improvement were the updates of clinical diagnoses and detection of previously missed variants, such as structural or mitochondrial variants, filtered or not captured variants (especially in the repetitive sequences, such as *RPGR* ORF15), or nonessential splice site variants. Consequently, we advocate for the inclusion of mitochondrial variant analysis and mobile element insertion assessment in routine ES analysis. Furthermore, we recommend against using interval files during variant calling, as there is a possibility of discovering pathogenic variants up to 150 bp beyond exon-intron junctions. Additionally, a targeted resequencing approach and deep phenotyping should be used on a case-by-case basis to identify hidden genetic variants.

Previous researchers have highlighted the advantages of ES reanalysis, which can increase diagnostic rates by 10% to 20%. This improvement is largely associated with the identification of new gene-disease associations and detection of CNVs that were missed in initial analyses.^8,9,10,11,39,40^ Consequently, reanalysis of ES is a potent and cost-efficient strategy for enhancing patient care.^9^ The clinician’s role is crucial in the success of ES reanalysis, as the ordering physician can contribute to the discovery of new disease-gene relationships and updated phenotypic information. Regardless of whether it is clinician or laboratory-driven, the identification of disease-causing variants continues to be a challenge due to incomplete phenotypic information, limited bioinformatic analysis, or insufficient disease association.^41^ In this study, the clinician-led reanalysis of ES was associated with a range of improvements: updated bioinformatic pipelines, patient phenotype evaluations, and recently reported genetic findings.

In our study, CNVs were responsible for molecular diagnoses in 6.4% of patients, with highest occurrence noted in *EYS*. These results were consistent with previous studies.^39,42^ We identified new single-exon CNVs in 3 patients. It is worth noting that previous studies reported that approximately 22% to 42% of CNVs involve a single exon.^6,43^ However, identifying single exon CNVs from ES data is particularly challenging, which is also reflected in the new release of the gnomAD version 4 data, in which sensitivity and specificity for CNVs covering fewer than 3 exons is low. Therefore, comprehensive CNV analysis with several different algorithms or additional high-depth targeted panel sequencing might be required to detect causal single-exon CNVs in IRDs.

The reasons for missed variants in the initial analysis may have included absence of genes in the analysis set, mitochondrial variants, MEIs, CNVs, and deep intronic variants. To address these issues, we recommend regularly updating the gene set for analysis, improving the bioinformatic pipeline to include MEIs and CNVs, and extending the analysis region to include area more than 150 bp from exon-intron boundaries. Although the mitochondrial regions were not originally designed to be captured by ES, off-target reads were sufficient for detection of variants in mitochondrial DNA,^44^ and the heteroplasmic level correlated well with the subsequent GS validation. Given the sufficient coverage of mitochondrial DNA in the ES kits and the fact that disease presentation alone is not sufficient to estimate the autosomal or mitochondrial inheritance, mitochondrial regions should be included in the analysis. Because coverage of the mitochondrial genome varies among exome capture kits (eFigure 13 in Supplement 1), clinicians should be aware of the limitations in mitochondrial variant calling using ES.

Reanalysis of sequencing data and variant interpretation is a challenging process. ES reanalysis entails revisiting previously generated data with updated annotation databases or new tools to detect variants that might have been missed during the initial analysis.^45^ In this study, the raw NGS data reprocessing, variant annotation, and interpretation were performed by an ophthalmologist who was also involved in the medical care of patients with IRD. This comprehensive analysis included clinical data reevaluation, detection of structural variants, Sanger sequencing of low-complexity repetitive regions, and functional minigene splicing assays. They all contributed to the increased diagnostic yield. Therefore, before moving toward GS, clinician-centered ES reanalysis with additional sequencing in the context of focused approach on phenotypically specified gene might be helpful to uncover hidden variants. Future research regarding automated exome reanalysis with a machine learning approach will be needed to improve diagnostic outcomes in Mendelian disorders.^8^

### Limitations

This study has several limitations. Our cohort consisted solely of Korean individuals, which may lead to different results in other racial and ethnic groups and introduce selection bias. Additionally, due to the nature of ES data, the sensitivity and specificity of CNV detection are lower compared with GS. The study was further constrained by lack of parental data for some patients. Furthermore, predicting the pathogenicity of variants is challenging, resulting in many variants being classified as VUS.

## Conclusions

In this cohort study, we concluded that the increased diagnostic yield was primarily associated with 2 factors: the identification of variants that were previously either filtered out or undetected, including MEIs, CNVs, mitochondrial variants, and deep intronic variants located beyond 150 bp from the exon-intron boundary, and a lack of established connections between certain genes and diseases at the time of the initial report. Consequently, using targeted resequencing in regions of low complexity, expanding the analytical range in ES without restrictions, analyzing both CNVs and MEIs, and detecting mitochondrial variants through ES may lead to new molecular diagnoses.

## Notes

Supplement 1. **eAppendix 1.** Exome Reanalysis **eAppendix 2.** Minigene Splicing Assay **eAppendix 3.** *RAB28* Wildtype and Altered Gblock **eAppendix 4.** *CNGB1* Wildtype and Altered Gblock **eAppendix 5.** List of Genes Associated With Inherited Retinal Diseases **eAppendix 6.** Initial Bioinformatic Analysis: Annotation, Interpretation of Variants, Phenotype Review, and Consensus Discussion **eTable 1.** General Features of the Cohort **eTable 2.** The Causative Variants Identified in 188 Patients With Inherited Retinal Diseases **eTable 3.** Novel Disease-Causing Variants, In Silico Predictions, and Minor Allele Frequencies **eTable 4.** Splicing Variants, In Silico Predictions, and Minor Allele Frequencies **eTable 5.** Copy Number Variations and ACMG/ClinGen Score of Copy Number Variations in 16 Patients **eFigure 1.** Schematic Diagram of Next-Generation Sequencing Analysis Workflow **eFigure 2.** Workflow of Copy Number Variations Analysis in Exome Sequencing Reanalysis **eFigure 3.** Schematic Presentation of High-Throughput Splicing Assay Vector for Minigene Splicing Assay **eFigure 4.** Manual Adjustment American College of Medical Genetics/American Molecular Pathology Criteria by Searching All Available Sources **eFigure 5.** The Distribution of Age at the Time of Genetic Testing **eFigure 6.** Distribution of Age of Onset and Total Number of Cases According to Causative Genes **eFigure 7.** The Segregation Analysis in Patients With Compound Heterozygous Variants **eFigure 8.** Frequent Altered Genes in this Cohort **eFigure 9.** New Molecular Diagnoses Made Based on Update of Clinical Diagnosis **eFigure 10.** Exome Sequencing Reanalysis Identified Hidden Structural Variants in Unsolved Patients With Inherited Retinal Diseases **eFigure 11.** Exome Sequencing Reanalysis and Long-Range PCR RPGR or f15 Sanger Sequencing Revealed Pathogenic Variants in Repetitive Difficult-to-Map Regions **eFigure 12.** Exome Sequencing Reanalysis With Functional Minigene Splicing Assay Identified a 3’ Exon Truncating Synonymous Variant **eFigure 13.** Mitochondrial Coverage According to Each Exome Kit Used in this Study
eReferences.

Supplement 2.
Data Sharing Statement
