# Supplementary material for: Clinician-Driven Reanalysis of Exome Sequencing Data From Patients With Inherited Retinal Diseases
Source: JAMA Netw Open. 2024 May 31;7(5):e2414198. doi: 10.1001/jamanetworkopen.2024.14198 (PMC11143468; doi:10.1001/jamanetworkopen.2024.14198)
Supplement: Supplement 1. — eAppendix 1. Exome Reanalysis eAppendix 2. Minigene Splicing Assay eAppendix 3. RAB28 Wildtype and Altered Gblock eAppendix 4. CNGB1 Wildtype and Altered Gblock eAppendix 5. List of Genes Associated With Inherited Retinal Diseases eAppendix 6. Initial Bioinformatic Analysis: Annotation, Interpretation of Variants, Phenotype Review, and Consensus Discussion eTable 1. General Features of the Cohort eTable 2. The Causative Variants Identified in 188 Patients With Inherited Retinal Diseases eTable 3. Novel Disease-Causing Variants, In Silico Predictions, and Minor Allele Frequencies eTable 4. Splicing Variants, In Silico Predictions, and Minor Allele Frequencies eTable 5. Copy Number Variations and ACMG/ClinGen Score of Copy Number Variations in 16 Patients eFigure 1. Schematic Diagram of Next-Generation Sequencing Analysis Workflow eFigure 2. Workflow of Copy Number Variations Analysis in Exome Sequencing Reanalysis eFigure 3. Schematic Presentation of High-Throughput Splicing Assay Vector for Minigene Splicing Assay eFigure 4. Manual Adjustment American College of Medical Genetics/American Molecular Pathology Criteria by Searching All Available Sources eFigure 5. The Distribution of Age at the Time of Genetic Testing eFigure 6. Distribution of Age of Onset and Total Number of Cases According to Causative Genes eFigure 7. The Segregation Analysis in Patients With Compound Heterozygous Variants eFigure 8. Frequent Altered Genes in this Cohort eFigure 9. New Molecular Diagnoses Made Based on Update of Clinical Diagnosis eFigure 10. Exome Sequencing Reanalysis Identified Hidden Structural Variants in Unsolved Patients With Inherited Retinal Diseases eFigure 11. Exome Sequencing Reanalysis and Long-Range PCR RPGR or f15 Sanger Sequencing Revealed Pathogenic Variants in Repetitive Difficult-to-Map Regions eFigure 12. Exome Sequencing Reanalysis With Functional Minigene Splicing Assay Identified a 3’ Exon Truncating Synonymous Variant eFigure 13. Mitochondrial Coverage Accor [file jamanetwopen-e2414198-s001.pdf]

Supplemental Online Content

Surl D, Won D, Lee S, et al. Clinician-driven reanalysis of exome sequencing data from patients with inherited retinal diseases. *JAMA Netw Open*. 2024;7(5):e2414198. doi:10.1001/jamanetworkopen.2024.14198

eAppendix 1. Exome Reanalysis
eAppendix 2. Minigene Splicing Assay
eAppendix 3. RAB28 Wildtype and Altered Gblock
eAppendix 4. CNGB1 Wildtype and Altered Gblock
eAppendix 5. List of Genes Associated With Inherited Retinal Diseases
eAppendix 6. Initial Bioinformatic Analysis: Annotation, Interpretation of Variants, Phenotype Review, and Consensus Discussion
eTable 1. General Features of the Cohort
eTable 2. The Causative Variants Identified in 188 Patients With Inherited Retinal Diseases
eTable 3. Novel Disease-Causing Variants, In Silico Predictions, and Minor Allele Frequencies
eTable 4. Splicing Variants, In Silico Predictions, and Minor Allele Frequencies
eTable 5. Copy Number Variations and ACMG/ClinGen Score of Copy Number Variations in 16 Patients
eFigure 1. Schematic Diagram of Next-Generation Sequencing Analysis Workflow
eFigure 2. Workflow of Copy Number Variations Analysis in Exome Sequencing Reanalysis
eFigure 3. Schematic Presentation of High-Throughput Splicing Assay Vector for Minigene Splicing Assay
eFigure 4. Manual Adjustment American College of Medical Genetics/American Molecular Pathology Criteria by Searching All Available Sources
eFigure 5. The Distribution of Age at the time of Genetic Testing
eFigure 6. Distribution of Age of Onset and Total Number of Cases According to Causative Genes
eFigure 7. The Segregation Analysis in Patients With Compound Heterozygous Variants
eFigure 8. Frequent Altered Genes in this Cohort
eFigure 9. New Molecular Diagnoses Made Based on Update of Clinical Diagnosis
eFigure 10. Exome Sequencing Reanalysis Identified Hidden Structural Variants in Unsolved Patients With Inherited Retinal Diseases
eFigure 11. Exome Sequencing Reanalysis and Long-Range PCR RPGR or f15 Sanger Sequencing Revealed Pathogenic Variants in Repetitive Difficult-to-Map Regions
eFigure 12. Exome Sequencing Reanalysis With Functional Minigene Splicing Assay Identified a 3' Exon Truncating Synonymous Variant
eFigure 13. Mitochondrial Coverage According to Each Exome Kit Used in this Study
eReferences.

This supplemental material has been provided by the authors to give readers additional information about their work.

© 2024 Surl D et al. *JAMA Network Open.*

eAppendix 1. Exome re-analysis

The fastq files were mapped to hg38 alt-masked references using dragmapper v1.3.0 (https://github.com/Illumina/DRAGMAP). And markduplicate reads, calibratedragstr and haplotypecaller with GATK was done with v.4.4.0.0. And then, the genomicsDB were generated with genomic VCF files, and joint calling were performed with VQSR. The joint VCF files were uploaded into seqr system (seqr.broadinstitute.org) and annotated with Variant Effect Prediction (VEP) tools.[1] To maximize calling variant, we also include mitochondrial regions in the interval file when we produced genomicsDB. Copy number variations (CNV) were called using ExomeDepth, cn.Mops, and GATK gCNV.[2-4] The CNV calling were annotated with annovar. The mobile element insertion (MEI) was called using SCRAMble and *RP1* grep programs.[5,6] The single nucleotide polymorphism (SNP) and small indels in known inherited eye diseases genes were reviewed, and identified new molecular diagnosis in 13 patients. The CNV variation analysis revealed new molecular diagnosis in 4 patients, and the MEI analysis identified *RP1* alu insertion in 5 patients. All SNP and small indel variants were classified with Amefican College of Medical Genetics (ACMG) criteria using Franklin by genoox (https://franklin.genoox.com/clinical-db/home).

© 2024 Surl D et al. *JAMA Network Open.*

eAppendix 2. Minigene Splicing Assay

The candidate mis-splicing deep intronic variant (*RAB28* 76-158T>G) c. and exon skipping variant [*CNGB1*: c.2154C>T:p.(Gly718=)] were selected, and gblock containing *AgeI* and *NheI* restriction sites were produced. Using our high-throughput splicing assay (HTSA) vector,[7] the gblock products were cloned into HTSA vector and used to transform chemically competent *Escherichia coli* (One Shot TOP10, Thermo Fisher, Waltham, MA). Plasmid DNA from single colonies was extracted with miniprep kits (ZymoPURE, Zymo Research) and analyzed by restriction enzyme digestion with *AgeI* and *NheI*. Human embryonic kidney (HEK293T) cells purchased from American Type Culture Collection (ATCC, Manassas, VA) were maintained in RPMI medium supplemented with 10% fetal bovine serum 1640 (Thermo Fisher). Then, 2 ml of 5×10^5 cells/ml were plated into each well of a 6-well plate (Corning Inc., Corning, NY) 12 h prior to transfections; 1–5 µg of vector DNA per well was used for transfections using a commercial reagent (Lipofectamine 2000, Invitrogen, Carlsbad, CA). Cells were harvested for RNA extraction 48 h after transfection. Cells were lysed with TRIzol (Thermo Fisher). After 1-Bromo-3-chloropropane or chloroform (Sigma-Aldrich, St. Louis, MO) treatment, the aqueous phase was transferred to mRNeasy columns with DNase I digestion performed on-column (Qiagen). Quantification was performed via NanoDrop (Thermo Fisher) and 500ng of RNA was converted to complementary DNA (cDNA) using oligo(dT) primers and SuperScript II (Thermo Fisher).

The mutant, control, and wild-type vectors described above were transfected into HEK293T. Two days after transfection, cDNA was generated as described and reverse transcription polymerase chain reaction (RT-PCR) was performed to amplify the flanking exons of the region of interest. The PCR products were purified by DNA-clean and concentrator kits (Zymo Research). DNA was extracted from gel bands via the ZymocleanTM Gel DNA Recovery Kit (Zymo Research, Irvine, CA) that were then Sanger sequenced.

© 2024 Surl D et al. *JAMA Network Open.*

eAppendix 3. *RAB28* wildtype and mutant (c.76-158T>G) gblock

>wildtype_RAB28 (exon 2: 97bp)
5'CTATCTATATATAGCTATCTATGTCTACCGGTTTATTTTTTAGTTTTTTTTTCTGTTGATAAC
TCTCAAATTCTAATTTTCAGCAAGGAAAAGGATCTTGACAAGCAGGGGATTTAAAGAGATCTA
ACTCATTAGTTCGTTGTCCCTACCGATAGCTGCTGGCAAGTAAAATTTACCTGGTGGTGTTAAG
TCTCTCCCCTCCTTGTAACTGATTAGAACTGCTTAAATTTGCTTGTTGGATTAATTGCAATATAC
ATGTTAAGATTGCTTAAGATTTATAGTGGTCTTATGATTACATTTCTTTAATGAATTAATTTTGAC
ATTCTGTGCATCAATTTTTTAGACCTCCTTAACTACGTGTTTTGCTCAAGAAACTTTTGGGAAA
CAGTACAAACAAACTATAGGACTGGATTTCTTTTTGAGAAGGATAACATTGCCAGGTAAGAGA
CTAAAAAGTCTTAACGTAGAAGATTATAAAAAAAATCTTAACAATAAG**GCTAGCGCAAGAGTT
CCAGCCGGGCTATTTAC3'**

>mutant_RAB28 (cryptic exon: 102bp, exon 2: 97bp)
5'CTATCTATATATAGCTATCTATGTCTACCGGTTTATTTTTTAGTTTTTTTTTCTGTTGATAAC
TCTCAAATTCTAATTTTCAGCAAGGAAAAGGATCTTGACAAGCAGGGGATTTAAAGAGATCTA
ACTCATTAGTTCGTTGTCCCTACCGATAGCTGCTGGCAAGTAAAATTTACCTGGTGGTGGTAAG
TCTCTCCCCTCCTTGTAACTGATTAGAACTGCTTAAATTTGCTTGTTGGATTAATTGCAATATAC
ATGTTAAGATTGCTTAAGATTTATAGTGGTCTTATGATTACATTTCTTTAATGAATTAATTTTGAC
ATTCTGTGCATCAATTTTTTAGACCTCCTTAACTACGTGTTTTGCTCAAGAAACTTTTGGGAAA
CAGTACAAACAAACTATAGGACTGGATTTCTTTTTGAGAAGGATAACATTGCCAGGTAAGAGA
CTAAAAAGTCTTAACGTAGAAGATTATAAAAAAAATCTTAACAATAAG**GCTAGCGCAAGAGTT
CCAGCCGGGCTATTTAC3'**

Sky blue highlight and bold = *AgeI*/*NheI* restriction enzyme site; green highlight = *RAB28* exon 2 and cryptic exon; yellow highlight and red = mutant site.

© 2024 Surl D et al. *JAMA Network Open.*

eAppendix 4. *CNGB1* wildtype and mutant (c.2154C>T) gblock

>wildtype_*CNGB1* (actual exon 21: 209bp)

5’CTATCTATATATAGCTATCTATGTCTACCGGTGTGCCACATGTAGTAGTTGAAGGAGTAGA
AAGAACAAAACCCTACCTGATCCAACTGGTGAAGAGCCTTGCCTGGCTGATGCTCTAGCGTC
CCCGTCAACCTTCCCCACAGACCTGATGTATGTCCTATGGCTGTTCTTCGTGGTGATGGCCTGG
AATTGGAACTGTTGGCTGATTCCCGTGCGCTGGGCCTTCCCCTACCAGACCCCGGACAACATC
CACCACTGGCTGCTGATGGATTACCTATGCGACCTCATCTACTTCCTGGACATCACCGTGTTCC
AGACACGCCTGCAGTTTGTCAGAGGCGGGGACATCATTGTGAGTCACAGGCAGGTGGTGTTG
GGGTGACCGGGGGCAGACCGCTCAGCCAGAGCCTCCAGGGCACTGACCCCTGATGCTGTCAT
AGATGACAAAATAAGTGAATAAGGAAATCTCAAGAACGGCTGCTGTGCTTGTTCAATAGCATG
GTTGTTTATTTTGCATGCCTTTGATACGTTCAAATTACGCACATCAGATATTGCTCATAGGTGAA
ATAGTTCCTGGACCACTTGACAGAATCAAAGATAGAGAAGTGGTCCAGGGAATGGTCTTTAGT
GAGGATGTACCTGGTTTGAATCCCACCTCCACCACAACTAGCTGAATCGCCTCTGTTTCCTCAT
CTGTAGAATGGGGCTAGTAATAGCTTCCACCTCCCAGTGTTGCCATGAGGATTGGTTGAAACG
ATGCATGCGGGGCGCTCAGCACAGGTATCCACTGGCAATTGACTTCTCCCAGGGGTCCTGCAA
AAACACACTGATGCTCTCCTACTTGGGCCAATTTGAGACCCTG**GCTAGCGCAAGAGTTCCA
GCCGGGCTATTTAC3'**

>mutant *CNGB1* c.2154C>T (predicted truncated exon 21: 195bp)

5’CTATCTATATATAGCTATCTATGTCTACCGGTGTGCCACATGTAGTAGTTGAAGGAGTAGA
AAGAACAAAACCCTACCTGATCCAACTGGTGAAGAGCCTTGCCTGGCTGATGCTCTAGCGTC
CCCGTCAACCTTCCCCACAGACCTGATGTATGTCCTATGGCTGTTCTTCGTGGTGATGGCCTGG
AATTGGAACTGTTGGCTGATTCCCGTGCGCTGGGCCTTCCCCTACCAGACCCCGGACAACATC
CACCACTGGCTGCTGATGGATTACCTATGCGACCTCATCTACTTCCTGGACATCACCGTGTTCC
AGACACGCCTGCAGTTTGTCAGAGGTGGGGACATCATTGTGAGTCACAGGCAGGTGGTGTTG
GGGTGACCGGGGGCAGACCGCTCAGCCAGAGCCTCCAGGGCACTGACCCCTGATGCTGTCAT
AGATGACAAAATAAGTGAATAAGGAAATCTCAAGAACGGCTGCTGTGCTTGTTCAATAGCATG
GTTGTTTATTTTGCATGCCTTTGATACGTTCAAATTACGCACATCAGATATTGCTCATAGGTGAA
ATAGTTCCTGGACCACTTGACAGAATCAAAGATAGAGAAGTGGTCCAGGGAATGGTCTTTAGT
GAGGATGTACCTGGTTTGAATCCCACCTCCACCACAACTAGCTGAATCGCCTCTGTTTCCTCAT
CTGTAGAATGGGGCTAGTAATAGCTTCCACCTCCCAGTGTTGCCATGAGGATTGGTTGAAACG
ATGCATGCGGGGCGCTCAGCACAGGTATCCACTGGCAATTGACTTCTCCCAGGGGTCCTGCAA
AAACACACTGATGCTCTCCTACTTGGGCCAATTTGAGACCCTG**GCTAGCGCAAGAGTTCCA
GCCGGGCTATTTAC**3'

Sky blue highlight and bold = *AgeI*/*NheI* restriction enzyme site; green highlight = *CNGB1* exon 21 and predicted truncated exon 21; yellow highlight and red = mutant site.

© 2024 Surl D et al. *JAMA Network Open.*

eAppendix 5. List of genes associated with inherited retinal diseases

AAAS, ABCA1, ABCA4, ABCB6, ABCD1, ABHD12, ACBD5, ACO2, ACOX1, ACP6, ACTB, ACTG1, ACVR1, ACVR2B, ADAM9, ADAMTS10, ADAMTS17, ADAMTS18, ADAMTSL4, ADGRA3, ADGRV1, ADIPOR1, AFG3L2, AGBL1, AGBL5, AGK, AGPS, AHDC1, AHI1, AHR, AIPL1, AIRE, AKAP9, AKR1E2, ALDH18A1, ALDH1A3, ALDH3A2, ALG2, ALG6, ALMS1, ALX1, AMACR, AMOT, AMT, ANKLE2, ANKS6, ANTXR1, AP3B1, AP3D1, APTX, ARHGEF10L, ARHGEF18, ARL13B, ARL2BP, ARL3, ARL6, ARMC9, ARNT2, ARSB, ARSG, ARX, ASB10, ASPA, ASPH, ASRGL1, ATAD3A, ATF4, ATF6, ATIC, ATM, ATOH7, ATP13A2, ATP1A3, ATP6V1A, ATP7B, ATP8A2, ATXN7, AUH, B3GALNT2, B3GLCT, B4GAT1, B9D1, B9D2, BBIP1, BBS1, BBS10, BBS12, BBS2, BBS4, BBS5, BBS7, BBS9, BCOR, BCS1L, BEST1, BFSP1, BFSP2, BLM, BLOC1S3, BLOC1S6, BMP1, BMP4, BMP7, BRAF, BRIP1, BTD, BUB1B, C12orf57, C19orf12, C1orf186, C1QTNF5, CA2, CABP4, CACNA1A, CACNA1F, CACNA1H, CACNA2D4, CACNB4, CANT1, CAPN15, CAPN5, CASK, CAV1, CBS, CC2D2A, CCDC103, CCDC28B, CCDC39, CCDC40, CCT2, CD320, CD96, CDH23, CDH3, CDHR1, CDKN2A, CDON, CEP104, CEP120, CEP164, CEP19, CEP250, CEP290, CEP41, CEP78, CEP83, CERKL, CFAP20, CFAP410, CFAP418, CFAP57, CHD3, CHD7, CHM, CHMP4B, CHN1, CHRDL1, CHST14, CHST6, CHSY1, CIB2, CISD2, CKAP4, CLCC1, CLCN7, CLDN19, CLK2, CLN3, CLN5, CLN6, CLN8, CLPB, CLRN1, CLUAP1, CNBP, CNGA1, CNGA3, CNGB1, CNGB3, CNNM4, CNTNAP2, COL11A1, COL11A2, COL17A1, COL18A1, COL1A1, COL1A2, COL25A1, COL2A1, COL4A1, COL4A3, COL4A4, COL4A5, COL5A1, COL5A2, COL8A2, COL9A1, COL9A2, COL9A3, COLEC11, COQ5, COX14, COX6B1, COX7B, CP, CPAMD8, CPLANE1, CRB1, CREBBP, CRELD1, CRIPT, CRPPA, CRTAP, CRX, CRYAA, CRYAB, CRYBA1, CRYBA4, CRYBB1, CRYBB2, CRYBB3, CRYGC, CRYGD, CRYGS, CSPP1, CTC1, CTCF, CTDP1, CTNNA1, CTNNB1, CTNS, CTSA, CTSD, CTSF, CWC27, CYP1B1, CYP27A1, CYP4V2, CYP51A1, CYTH3, DAG1, DBH, DCAF6, DCDC1, DCDC2, DCN, DCT, DDB2, DDX11, DDX58, DHCR7, DHDDS, DHODH, DHX38, DIAPH1, DLG4, DMD, DNAAF1, DNAAF11, DNAAF2, DNAAF3, DNAAF5, DNAH10, DNAH11, DNAH5, DNAH9, DNAI1, DNAI2, DNAJC17, DNAJC19, DNAJC30, DNAJC5, DNAL1, DNM1L, DNM2, DNMT3B, DOCK6, DPAGT1, DPM1, DPP6, DRAM2, DSE, DTHD1, DTNBP1, DYNC2H1, DYNC2I2, EBF3, EBP, EDN3, EDNRA, EFEMP1, EIF4G1, ELOVL1, ELOVL4, ELP4, EMC1, EPG5, EPHA2, ERCC1, ERCC2, ERCC3, ERCC4, ERCC5, ERCC6, ERCC8, ESCO2, ESPN, EVC, EVC2, EXOSC2, EXOSC8, EYA1, EYS, FAM111A, FAM126A, FAM161A, FANCA, FANCB, FANCC, FANCD2, FANCE, FANCF, FANCG, FANCI, FANCL, FANCM, FAR1, FASTKD2, FAT1, FBLN5, FBN1, FBN2, FBXL4, FDXR, FGFR1, FIBP, FKBP14, FKRP, FKTN, FLNA, FLNB, FLVCR1, FOXC1, FOXC2, FOXD1, FOXD3, FOXE3, FOXF2, FOXH1, FOXL2, FOXO1, FOXRED1, FRAS1, FREM1, FREM2, FRMD7, FSCN2, FTL, FXN, FYCO1, FZD4, GABRB2, GALE, GALK1, GALM, GALNS, GALT, GBA, GBA2, GCNT2, GCSH, GDF1, GDF3, GDF6, GFER, GJA1, GJA3, GJA8, GJB2, GJB6, GJC2, GJE1, GLA, GLB1, GLDC, GLIS2, GLIS3, GM2A, GMPPA, GMPPB, GNAT1, GNAT2, GNB1, GNB3, GNPAT, GNPTG, GNS, GPHN, GPR143, GPR179, GRIN1, GRIN2B, GRIP1, GRK1, GRM6, GRN, GSN, GTF2H5, GUCA1A, GUCA1B, GUCY2D, GUSB, HADHA, HADHB, HARS1, HCCS, HCN1, HDAC6, HESX1, HEXA, HEXB, HGSNAT, HK1, HKDC1, HLCS, HMCN1, HMGB3, HMX1, HNRNPDL, HOXA1, HOXB1, HPS1, HPS3, HPS4, HPS5, HPS6, HSF4, HYLS1, IARS2, IBA57, IDH3A, IDH3B, IDUA, IFIH1, IFT122, IFT140, IFT172, IFT27, IFT43, IFT52, IFT74, IFT80, IFT81, IFT88, IGBP1, IGFBP7, IMPDH1, IMPG1, IMPG2, INPP5E, INVS, IQCB1, IRX1, ISCA2, ITM2B, ITPR1, JAG1, JAM3, KAT6B, KATNIP, KCNA1, KCNJ12, KCNJ13, KCNJ3, KCNQ3, KCNV2, KCTD19, KCTD7, KDM6A, KERA, KIAA0586, KIAA0753, KIAA1549, KIDINS220, KIF11, KIF21A, KIF3B, KIF5A, KIF7, KIZ, KLF10, KLHL7, KMT2D, KRT12, KRT2, KRT3, LAMA1, LAMB1, LAMB2, LARGE1, LCA5, LCAT, LCT, LEFTY2, LEMD2, LHX2, LIM2, LMX1B, LONP1, LOXHD1, LOXL1, LRAT, LRIT3, LRMDA, LRP2, LRP5, LRPAP1, LSS, LTBP2, LTBP3, LYST, LZTFL1, MAB21L2, MAF, MAFB, MAK, MAN2B1, MAP3K7, MAPKAPK3, MARK3, MBD5, MBTPS2, MC1R, MCEE, MCOLN1, MECR, MED25, MERTK, MFF, MFN2, MFRP, MFSD6L, MFSD8, MICALCL, MIEF1, MIP, MIPEP, MIR184, MIR204, MITF, MKKS, MKS1, MLPH, MMAA, MMAB, MMACHC, MMADHC, MMP1, MMP19, MMUT, MOCS1, MPDU1, MPLKIP, MRE11, MSMO1, MT-ATP6, MT-CYB, MT-ND1, MT-ND4, MT-ND6, MT-TL1, MT-TS2, MTO1, MTPAP, MTRFR, MTTP, MVK, MYF5, MYH9, MYO5A, MYO7A, MYOC, NAA10, NBAS, NDP, NDUFA1, NDUFA10, NDUFA11, NDUFA12, NDUFA2, NDUFA9, NDUFAF1, NDUFAF2, NDUFAF3, NDUFAF4, NDUFAF5, NDUFAF6, NDUFB3, NDUFS1, NDUFS2, NDUFS3, NDUFS4, NDUFS6, NDUFS7, NDUFS8, NDUFV1, NDUFV2, NECTIN3, NEK1, NEK2, NEK4, NEK8, NEU1, NEUROD1, NF1, NF2, NGLY1, NHS, NIM1K, NKX2-5, NLRP1, NLRP3, NME8, NMNAT1, NOD2, NODAL, NOTCH2, NPC1, NPHP1, NPHP3, NPHP4, NR2E3, NR2F1, NRL, NSD1, NSUN3, NT5C2, NTF4, NUB1, NUS1, NXNL1, NYX, OAT, OCA2, OCRL, ODAD1, OFD1, OPA1, OPA3, OPN1LW, OPN1MW, OPN1SW, OPTN, OSMR, OSTM1, OTX2, OVOL2, P3H2, P4HA2, PANK2, PAX2, PAX3, PAX6, PCARE, PCDH15, PCNA, PCYT1A, PDE6A, PDE6B,

© 2024 Surl D et al. *JAMA Network Open.*

PDE6C, PDE6D, PDE6G, PDE6H, PDHA1, PDSS1, PDZD7, PET100, PEX1, PEX10, PEX11B, PEX12, PEX13, PEX14, PEX16, PEX19, PEX2, PEX26, PEX3, PEX5, PEX5L, PEX6, PEX7, PFKM, PGAP1, PGK1, PHGDH, PHOX2A, PHYH, PIEZO2, PIGL, PIGT, PIGY, PIK3R1, PIKFYVE, PITPNM3, PITX2, PITX3, PLA2G5, PLA2G6, PLG, PLK4, PLOD1, PLOD3, PLP1, PMM2, PNPLA6, POC1B, POC5, POGZ, POLA1, POLG, POMGNT1, POMGNT2, POMK, POMT1, POMT2, PORCN, PPT1, PQBP1, PRCD, PRDM13, PRDM5, PRIMPOL, PRKAA2, PRKACA, PRKCG, PROM1, PRPF3, PRPF31, PRPF4, PRPF6, PRPF8, PRPH2, PRPS1, PRSS56, PTCH1, PTF1A, PTPN11, PTPN23, PXDN, PYGM, RAB18, RAB27A, RAB28, RAB3GAP1, RAB3GAP2, RAD50, RAI1, RARB, RAX, RAX2, RB1, RBP3, RBP4, RCBTB1, RD3, RDH11, RDH12, RDH5, RECQL4, REEP6, RERE, RGR, RGS9, RGS9BP, RHEX, RHO, RIMS2, RIPK4, RLBP1, RNLS, RNU4ATAC, ROBO3, ROM1, RP1, RP1L1, RP2, RP9, RPE65, RPGR, RPGRIP1, RPGRIP1L, RPS19, RRM2B, RS1, RSPH4A, RSPH9, RSPO1, RTN4IP1, RXYLT1, SACS, SAG, SALL2, SALL4, SAMD11, SAMD9, SC5D, SCAPER, SCARF2, SCLT1, SCN8A, SCO2, SDCCAG8, SDHAF1, SEC23A, SEMA3E, SEMA4A, SEMA6A, SERPINH1, SETX, SH3PXD2B, SHH, SIL1, SIPA1L3, SIX3, SIX5, SIX6, SLC16A12, SLC1A1, SLC1A3, SLC24A1, SLC24A5, SLC25A1, SLC25A16, SLC25A46, SLC25A5, SLC2A1, SLC33A1, SLC35A2, SLC35A4, SLC38A8, SLC39A5, SLC45A2, SLC4A11, SLC4A4, SLC4A5, SLC4A7, SLC52A2, SLC52A3, SLC66A1, SLC6A6, SLC7A14, SLITRK6, SLX4, SMCHD1, SNRNP200, SNX10, SNX3, SOD1, SON, SOX2, SOX3, SOX5, SPARC, SPATA7, SPG7, SPINT2, SPP2, SPTBN2, SRD5A3, SREBF2, SSBP1, ST3GAL5, STIM1, STK38L, STN1, STRA6, STS, STT3B, SUCLA2, SUOX, TACSTD2, TAT, TBC1D20, TBC1D7, TBCE, TBK1, TBL1XR1, TCF4, TCIRG1, TCOF1, TCTN1, TCTN2, TCTN3, TDRD7, TEAD1, TEK, TENM1, TENM3, TFAP2A, TGFBI, TIMM8A, TIMP3, TINF2, TK2, TLR3, TLR4, TMEM107, TMEM114, TMEM126A, TMEM138, TMEM216, TMEM231, TMEM237, TMEM67, TMEM70, TMEM98, TMTC3, TNFSF11, TNPO1, TOPORS, TP63, TPP1, TRAF3IP1, TREX1, TRIM32, TRIM37, TRIM44, TRNT1, TRPC1, TRPM1, TRPM2, TSC1, TSC2, TSFM, TSPAN12, TTC21B, TTC8, TTLL5, TTPA, TTR, TUB, TUBA8, TUBB, TUBB3, TUBGCP4, TUBGCP6, TULP1, TWNK, TYMP, TYR, TYRP1, UBE3B, UBIAD1, UBR1, UCHL1, UFSP2, UHMK1, UNC119, UNC45B, USH1C, USH1G, USH2A, USP45, USP9X, VAX1, VCAN, VIM, VPS13B, VSX1, VSX2, WAC, WASF3, WDPCP, WDR17, WDR19, WDR35, WDR36, WFS1, WHRN, WRN, WT1, XPA, XPC, XPNPEP3, YAP1, YME1L1, ZEB1, ZEB2, ZFP30, ZIC2, ZNF408, ZNF423, ZNF469, ZNF513, ZNF592, ZNF644, ZNHIT3

© 2024 Surl D et al. *JAMA Network Open.*

eAppendix 6. Initial bioinformatic analysis: Annotation, Interpretation of Variants, Phenotype Review, and Consensus Discussion

First (step 1), variants were filtered by their frequencies in population control databases, including Genome Aggregation Database, ESP6500, 1000 Genomes Project, and Korean Reference Genome DataBase. To maximize the diagnostic yield, variants with a minor allele frequency (MAF) greater than 5% in any of the population subgroups rather than conventional 1% criteria were classified as absolutely benign, whereas those that were absent from the general population were considered to have moderate evidence of being pathogenic. Secondly (step 2), literature and database searches for previous reports and functional studies were performed using the RetNet database, the Alamut Visual software and Human Gene Mutation Database professional database. Pathogenic or benign evidence was scored when predictions of all *in silico* algorithms agreed. Finally, the last step involved genetic specialists or laboratory physicians presenting a preliminary report to the patient's attending physicians or pediatric ophthalmologists, which listed all possible pathogenic variants, likely pathogenic variants, and variants of unknown significance (VUSs). When pathogenic or likely pathogenic variants were consistent with the patient's phenotype based on in-depth review by ophthalmologists, final validation using other confirmatory assays and a parental study was planned if available. VUSs, especially missense variants, were prioritized according to population frequency, American College of Medical Genetics score, and the patient's ocular phenotype. A parental study was scheduled to detect de novo occurrence of the candidate pathogenic or likely pathogenic variants, and VUSs in all available trios.

© 2024 Surl D et al. *JAMA Network Open.*

eTable 1. General features of the cohort

**Age of proband at age of genetic testing**		
< 10	28	10.6%
10-25	68	25.8%
26-50	107	40.5%
> 50	61	23.1%
**Sex**		
Male	151	57.2%
Female	113	42.8%
**Inheritance (based on family history)**		
Isolated	173	65.5%
Autosomal recessive	52	19.7%
Autosomal dominant	26	9.8%
X-linked or mitochondrial	13	4.9%
**Phenotype**		
Retinitis pigmentosa	129	48.9%
Stargardt/Macular dystrophy	26	9.8%
Cone rod/cone dystrophy	22	8.3%
LCA/EOSRD	21	8.0%
Optic atrophy	9	3.4%
FEVR	7	2.7%
Achromatopsia	7	2.7%
Choroideremia	4	1.5%
Bestrophinopathy	3	1.1%
Bardet-Biedl syndrome	3	1.1%
Others	33	12.5%

Abbreviations: EOSRD, early onset severe retinal dystrophy; FEVR, familial exudative vitreoretinopathy; LCA, Leber congenital amaurosis.

© 2024 Surl D et al. *JAMA Network Open.*

eTable 2. The disease causing variants identified in 188 patients with inherited retinal diseases.

Patient ID	Gene	Transcript	Nucleotide change	Amino acid change	CADD	FATHMM	gnomAD[a]	ACMG	Phase
SH_0001	*GPR143*	NM_000273.2	c.925del	p.(Ala309Profs*24)	34	0.929	.	P	No need
SB_0002	*ABCA4*	NM_000350.2	c.1933G>A<br>c.3470T>G	p.(Asp645Asn)<br>p.(Leu1157*)	28.4<br>43	0.965<br>0.996	5/251094<br>.	P<br>P	NC
SH_0003	*CNGA3*	NM_001298.2	c.829C>T<br>c.1001C>T	p.(Arg277Cys)<br>p.(Ser334Phe)	31<br>26	0.936<br>0.918	24/251350<br>.	LP<br>LP	Confirmed
SH_0005	*EYS*	NM_001142800.1	c.4957dup<br>c.6563T>C	p.(Ser1653Lysfs*2)<br>p.(Ile2188Thr)	35<br>23.4	0.853<br>0.956	1/10890<br>1/10762	P<br>LP	Confirmed
SH_0008	*GPR143*	NM_000273.2	c.703G>A	p.(Glu235Lys)	27.7	0.953	.	LP	No need
SH_0009	*EYS*	NM_001142800.1	c.4957dup<br>c.4957dup	p.(Ser1653Lysfs*2)<br>p.(Ser1653Lysfs*2)	35	0.853	1/151914	P	No need
SH_0010	*SLC45A2*	NM_016180.3	c.469G>A<br>c.686G>A	p.(Asp157Asn)<br>p.(Cys229Tyr)	31<br>24.6	0.986<br>0.888	1/251482<br>2/151458	LP<br>LP	Confirmed
SH_0011	*RPGR*	NM_001034853.1	c.2405_2406del	p.(Glu802Glyfs*32)	18.92	0.014	.	LP	No need
SH_0012	*PROM1*	NM_006017.2	c.1117C>T	p.(Arg373Cys)	15.09	0.042	1/249148	LP	No need
SH_0014	*ABCA4*	NM_000350.2	c.880C>T<br>c.4748T>C	p.(Gln294*)<br>p.(Leu1583Pro)	36<br>25.9	0.973<br>0.994	.<br>2/251476	P<br>LP	Confirmed
SH_0015	*RPE65*	NM_000329.2	c.1067dup<br>c.1543C>T	p.(Asn356Lysfs*9)<br>p.(Arg515Trp)	34<br>27.7	0.963<br>0.825	.<br>4/280264	P<br>P	Confirmed
SS_0016	*USH2A*	NM_206933.2	c.3745C>T<br>c.4957C>T	p.(Pro1249Ser)<br>p.(Arg1653*)	23.4<br>33	0.965<br>0.199	.<br>5/282008	LP<br>P	NC
SH_0017	*EYS*	NM_001142800.1	c.4957dup<br>c.6395T>C	p.(Ser1653Lysfs*2)<br>p.(Leu2132Pro)	35<br>26	0.853<br>0.895	1/151914<br>.	P<br>LP	NC
SH_0018	*RP1*	NM_006269.1	c.289G>T<br>c.4196del	p.(Glu97*)<br>p.(Cys1399Leufs*5)	35<br>22.1	0.966<br>0.826	.<br>1/250622	P<br>P	Confirmed
SS_0019	*USH2A*	NM_206933.2	c.1645-2A>C<br>c.2802T>G	.<br>p.(Cys934Trp)	34<br>25.5	0.989<br>0.974	.<br>57/282482	P<br>LP	NC
SH_0021	*RPGR*	NM_001034853.1	c.2779del	p.(Glu927Lysfs*162)	23.4	0.025	.	P	No need

© 2024 Surl D et al. *JAMA Network Open.*

SH_0022	*EYS*	NM_001142800.1	c.2528G>A<br>c.8868C>A	p.(Gly843Glu)<br>p.(Tyr2935*)	23.9<br>35	0.983<br>0.481	4/156432<br>7/188242	LP<br>LP	Infer by gnomAD[c]
SH_0024	*USH2A*	NM_206933.2	c.4758+3A>G<br>c.15233C>G	.<br>p.(Pro5078Arg)	20.7<br>26	0.988<br>0.996	422/281358<br>17/282846	LP<br>LP	Infer by gnomAD[c]
SH_0025	*EYS*	NM_001142800.1	c.4957dup<br>c.4957dup	p.(Ser1653Lysfs*2)<br>p.(Ser1653Lysfs*2)	35	0.853	1/151914	P	No need
SH_0029	*EYS*	NM_001142800.1	c.6557G>A<br>exon 4 deletion	p.(Gly2186Glu)<br>.	22.8<br>NA	0.815<br>NA	6/149126<br>.	LP<br>LP	NC
SS_0031	*PDE6B*	NM_000283.3	c.1604T>A<br>c.2068C>T	p.(Ile535Asn)<br>p.(Gln690*)	25.8<br>36	0.990<br>0.427	1/250482<br>.	LP<br>P	NC
SB_0032	*IMPG1*	NM_001563.4	c.1896T>G	p.(Ser632Arg)	23.7	0.882	.	VUS	No need
SH_0035	*PDE6C*	NM_006204.3	c.85C>T<br>c.1771G>A	p.(Arg29Trp)<br>p.(Glu591Lys)	24.1<br>28.1	0.899<br>0.987	6/282886<br>2/251120	LP<br>LP	Confirmed
SS_0037	*MAK*	NM_001242957.1	c.502C>G<br>c.553G>A	p.(Pro168Ala)<br>p.(Ala185Thr)	25.2<br>28.3	0.973<br>0.980	1/251388<br>2/251440	LP<br>LP	IGV confirmed
SH_0038	*PRPH2*	NM_000322.4	c.658C>T	p.(Arg220Trp)	25	0.818	.	LP	No need
SS_0039	*CHM*	NM_000390.4	exon 2-8 duplication	.	NA	NA	.	LP	No need
SH_0040	*NMNAT1*	NM_022787.3	c.196C>T<br>c.709C>T	p.(Arg66Trp)<br>p.(Arg237Cys)	31<br>35	0.980<br>0.974	22/282832<br>14/277114	P<br>P	Confirmed
SH_0041	*RP1*	NM_006269.1	c.4196del<br>c.5797C>T	p.(Cys1399Leufs*5)<br>p.(Arg1933*)	22.1<br>38	0.826<br>0.594	1/250622<br>49/281934	P<br>LP	Confirmed
SH_0042	*PAX2*	NM_000278.3	c.95C>G	p.(Pro32Arg)	29.2	0.989	.	LP	No need
SH_0044	*ABCA4*	NM_000350.2	c.1222C>T<br>c.3470T>G	p.(Arg408*)<br>p.(Leu1157*)	37<br>41	0.164<br>0.996	5/282850<br>.	LP<br>P	Confirmed
SH_0046	*EYS*	NM_001142800.1	c.2471G>A<br>c.4957dup	p.(Cys824Tyr)<br>p.(Ser1653Lysfs*2)	18.93<br>35	0.876<br>0.853	.<br>1/151914	LP<br>P	NC
SH_0050	*EYS*	NM_001142800.1	c.1185-6T>G<br>c.9340G>C	.<br>p.(Asp3114His)	22.5<br>24.4	0.988<br>0.975	1/246084<br>.	LP<br>LP	Confirmed
SH_0051	*CRX*	NM_000554.4	c.118C>T	p.(Arg40Trp)	26.8	0.814	1/250660	LP	No need

© 2024 Surl D et al. *JAMA Network Open.*

SH_0052	*BBS9*	NM_198428.2	c.1016+4del<br>exon 14-19 deletion	.<br>.	24.8<br>NA	0.984<br>NA	.<br>.	LP<br>LP	Confirmed
SH_0055	*EYS*	NM_001142800.1	c.7048del<br>c.9106_9107insTATA	p.(Cys2350Alafs*97)<br>p.(Asn3036Ilefs*2)	32<br>32	0.973<br>0.939	.<br>.	P<br>P	NC
SH_0056	*EYS*	NM_001142800.1	c.1989del<br>c.4957dup	p.(Tyr664Ilefs*19)<br>p.(Ser1653Lysfs*2)	15.52<br>35	0.566<br>0.853	.<br>1/151914	P<br>P	NC
SH_0058	*CNGB1*	NM_001297.4	c.217+5G>C<br>c.2154C>T	.<br>p.(Gly718=)	23.2<br>15.9	0.972<br>0.088	13/280826<br>12/248428	LP<br>LP	Infer by gnomAD[c]
SH_0059	*NBAS*	NM_015909.3	c.3494del<br>c.5740C>T	p.(Val1165Serfs*31)<br>p.(Arg1914Cys)	34<br>24.9	0.934<br>0.942	.<br>.	P<br>LP	Confirmed
SH_0060	*EYS*	NM_001142800.1	c.2471G>A<br>c.4957dup	p.(Cys824Tyr)<br>p.(Ser1653Lysfs*2)	18.93<br>35	0.876<br>0.853	.<br>1/151914	LP<br>P	Confirmed
SS_0061	*BBS10*	NM_024685.4	c.235dup<br>c.1391C>G	p.(Thr79fs*17)<br>p.(Ser464*)	24.4<br>31	0.739<br>0.260	2/240916<br>3/251346	P<br>P	Infer by gnomAD[c]
SS_0062	*RPGR*	NM_000328.2	c.310+1G>T	.	34	0.996	.	P	No need
SH_0063	*RP1*	NM_006269.2	c.2029C>T	p.(Arg677*)	33	0.705	.	P	No need
SH_0066	*RP1*	NM_006269.1	c.4052_4053ins328<br>c.5797C>T	p.(Tyr1352Alafs*9)<br>p.(Arg1933*)	NA<br>38	NA<br>0.594	.<br>49/281934	P<br>LP	Confirmed
SH_0067	*PRPF31*	NM_015629.3	exon 6-14 duplication	.	NA	NA	.	VUS	No need
GS_0001	*USH2A*	NM_206933.2	c.2802T>G<br>c.2802T>G	p.(Cys934Trp)<br>p.(Cys934Trp)	25.5	0.974	57/282482	LP	No need
GS_0003	*EYS*	NM_001142800.1	c.8805C>A<br>c.8963C>T	p.(Tyr2935*)<br>p.(Thr2988Ile)	35<br>23.8	0.481<br>0.966	7/188242<br>.	P<br>LP	IGV confirmed
GS_0004	*USH2A*	NM_206933.2	c.5678C>G<br>c.10973T>C	p.(Ser1893*)<br>p.(Leu3648Pro)	41<br>24.5	0.988<br>0.977	.<br>.	P<br>LP	NC
GS_0006	*EYS*	NM_001142800.1	c.4957dup<br>exon 4-5 deletion	p.(Ser1653Lysfs*2)<br>.	35<br>NA	0.853<br>NA	1/151914<br>.	P<br>P	NC
GH_0008	*BEST1*	NM_004183.3	c.632T>C<br>c.830C>T	p.(Leu211Pro)<br>p.(Thr277Met)	26.2<br>24.6	0.995<br>0.974	.<br>3/251476	LP<br>LP	Confirmed

© 2024 Surl D et al. *JAMA Network Open.*

GS_0010	*USH2A*	NM_206933.2	c.251G>A<br>c.14243C>T	p.(Cys84Tyr)<br>p.(Ser4748Phe)	23.2<br>28.8	0.914<br>0.995	.<br>1/251252	VUS<br>LP	NC
GS_0011	*RHO*	NM_000539.3	c.84G>C	p.(Gln28His)	25.3	0.962	.	LP	No need
GH_0012	*EYS*	NM_001142800.1	c.4957dup<br>exon 6-8 deletion	p.(Ser1653Lysfs*2)<br>.	35<br>NA	0.853<br>NA	1/151914<br>.	P<br>P	Confirmed
GH_0014	*NMNAT1*	NM_022787.3	c.196C>T<br>c.709C>T	p.(Arg66Trp)<br>p.(Arg237Cys)	31<br>29.1	0.98<br>0.974	21/246218<br>14/277114	P<br>P	Confirmed
GH_0015	*USH2A*	NM_206933.2	c.2802T>G<br>c.11389+3A>T	p.(Cys934Trp)<br>.	25.5<br>22.7	0.974<br>0.968	57/282482<br>2/250762	LP<br>LP	Infer by gnomAD[c]
GH_0016	*CEP290*	NM_025114.3	c.322C>T<br>c.1429C>T	p.(Arg108*)<br>p.(Arg477*)	34<br>36	0.864<br>0.905	1/248768<br>1/148408	P<br>P	Confirmed
GH_0017	*RPGR*	NM_001034853.1	c.518G>A	p.(Gly173Asp)	24.6	0.976	.	LP	No need
GH_0018	*PAX6*	NM_000280.4	chr 11p13 deletion	.	NA	NA	.	P	No need
GH_0019	*TYR*	NM_000372.4	c.1037-7T>A<br>c.1147G>A	.<br>p.(Asp383Asn)	18.81<br>31	0.246<br>0.989	242/280938<br>25/282406	LP<br>P	IGV confirmed
GH_0021	*RP1*	NM_006269.1	c.4052_4053ins328<br>c.4196del	p.(Tyr1352Alafs*9)<br>p.(Cys1399Leufs*5)	NA<br>22.1	NA<br>0.826	.<br>1/250622	P<br>P	Confirmed
GH_0022	*ABCA4*	NM_000350.2	c.664dup<br>c.6118C>T	p.(Ala222Glyfs*58)<br>p.(Arg2040*)	23.3<br>53	0.955<br>0.978	.<br>4/282710	LP<br>P	Confirmed
GS_0024	*USH2A*	NM_206933.2	c.2802T>G<br>c.12708T>A	p.(Cys934Trp)<br>p.(Cys4236*)	25.5<br>40	0.974<br>0.966	57/282482<br>.	P<br>P	NC
GH_0025	*PDE6C*	NM_006204.3	c.85C>T<br>c.827G>A	p.(Arg29Trp)<br>p.(Arg276Gln)	24.1<br>26.7	0.899<br>0.978	6/282886<br>5/282768	LP<br>LP	Confirmed
GH_0026	*CERKL*	NM_001030311.2	c.156_157insT<br>c.1283_1286dup	p.(Glu53*)<br>p.(Ile430Glnfs*14)	32<br>35	0.500<br>0.973	.<br>.	P<br>P	NC
GH_0027	*ABCA4*	NM_000350.2	c.1957C>T<br>c.5312G>T	p.(Arg653Cys)<br>p.(Gly1771Val)	32<br>35	0.993<br>0.987	4/248260<br>.	P<br>LP	Confirmed
GH_0028	*GPR143*	NM_000273.2	c.14G>C	p.(Arg5Pro)	27.7	0.921	.	LP	No need
GS_0029	*RP2*	NM_006915.2	c.100_102+17del	p.(Lys34del)	24.7	0.167	.	LP	No need
GH_0030	*CRB1*	NM_001257966.1	c.1576C>T<br>c.1577G>C	p.(Arg526*)<br>p.(Arg526Pro)	33<br>23	0.432<br>0.622	9/282816<br>.	P<br>LP	IGV confirmed

© 2024 Surl D et al. *JAMA Network Open.*

GH_0031	*USH2A*	NM_206933.2	c.486-14G>A<br>c.820C>T	.<br>p.(Arg274*)	17.49<br>38	0.975<br>0.951	5/250430<br>2/250882	LP<br>P	NC
GS_0032	*EYS*	NM_001142800.1	c.4957dup<br>c.4957dup	p.(Ser1653Lysfs*2)<br>p.(Ser1653Lysfs*2)	35	0.853	1/151914	P	No need
GH_0034	*KLHL7*	NM_001031710.2	c.449G>A	p.(Ser150Asn)	20.4	0.954	.	LP	No need
GH_0035	*USH2A*	NM_206933.2	c.8559-2A>G<br>c.13903C>T	.<br>p.(Gln4635*)	34<br>44	0.994<br>0.985	8/251134<br>.	LP<br>P	Confirmed
GS_0037	*RP1*	NM_006269.1	c.4052_4053ins328<br>c.5797C>T	p.(Tyr1352Alafs*9)<br>p.(Arg1933*)	NA<br>38	NA<br>0.594	.<br>49/281934	P<br>P	Confirmed
GS_0039	*KLHL7*	NM_001031710.2	c.433A>G	p.(Asn145Asp)	26.6	0.985	.	LP	No need
GH_0040	*CEP290*	NM_025114.3	c.4661_4663del<br>c.6012-12T>A	p.(1554_1555del)<br>.	21.6<br>23.3	0.959<br>0.252	7/280272<br>4/224124	LP<br>LP	Confirmed
GH_0041	*CC2D2A*	NM_001080522.2	c.934G>A<br>c.2803C>T	p.(Glu312Lys)<br>p.(Arg935*)	32<br>38	0.976<br>0.908	.<br>3/269734	LP<br>P	Confirmed
GH_0042	*CNGA1*	NM_001142564.1	c.398del<br>c.472del	p.(Gly133Valfs*29)<br>p.(Leu158Phefs*4)	18.8<br>29	0.029<br>0.958	.<br>29/280626	LP<br>LP	NC
GH_0044	*NMNAT1*	NM_022787.3	c.709C>T<br>exon 2 deletion	p.(Arg237Cys)<br>.	35<br>NA	0.974<br>NA	14/277114<br>.	LP<br>P	Confirmed
GH_0045	*ABCA4*	NM_000350.2	c.763C>T<br>c.6320G>A	p.(Arg255Cys)<br>p.(Arg2107His)	24.2<br>32	0.901<br>0.988	26/282798<br>573/282666	LP<br>LP	Confirmed
GS_0046	*KIZ*	NM_001276389.2	c.141_144del<br>c.141_144del	p.(Pro48Leufs*27)<br>p.(Pro48Leufs*27)	23.3	0.535	.	LP	No need
GH_0048	*ABCA4*	NM_000350.2	c.768G>A<br>c.880C>T	p.(Val256=)<br>p.(Gln294*)	23<br>36	0.967<br>0.973	.<br>.	LP<br>P	Confirmed
GH_0049	*ABCA4*	NM_000350.2	c.880C>T<br>c.3035_3037del	p.(Gln294*)<br>p.(Asn1012del)	36<br>22.8	0.973<br>0.972	.<br>1/251468	P<br>LP	Confirmed
GS_0050	*IMPDH1*	NM_000883.3	c.147-2A>G	.	24.5	0.389	.	LP	No need
GH_0051	*BBS1*	NM_024649.5	c.159G>C<br>exon 10-12 deletion	p.(Lys53Asn)<br>.	36<br>NA	0.967<br>NA	.<br>.	LP<br>P	Confirmed
GH_0053	*RHO*	NM_000539.3	c.937-1G>A	.	34	0.994	.	P	No need

© 2024 Surl D et al. *JAMA Network Open.*

GH_0054	*RAB28*	NM_001017979.2	c.68C>T<br>c.76-158T>G	p.(Ser23Phe)<br>.	26<br>12.13	0.666<br>0.171	7/219238<br>.	LP<br>LP	Confirmed
GS_0055	*EYS*	NM_001142800.1	c.6557G>A<br>c.6563T>C	p.(Gly2186Glu)<br>p.(Ile2188Thr)	22.8<br>23.1	0.815<br>0.956	6/149126<br>1/149652	LP<br>LP	IGV confirmed
GH_0058	*ABCA4*	NM_000350.2	c.1222C>T<br>c.3623T>G	p.(Arg408*)<br>p.(Leu1208Arg)	37<br>28.7	0.164<br>0.987	5/282850<br>.	P<br>LP	NC
GH_0059	*CHM*	NM_000390.2	c.403_404del	p.(Asp135Phefs*9)	22.2	0.755	.	P	No need
GH_0061	*CYP4V2*	NM_207352.3	c.675-1G>A<br>c.802-8_810del17insGC	.<br>exon 7 deletion	33<br>28.7	0.996<br>0.950	.<br>.	P<br>P	NC
GS_0063	*USH2A*	NM_206933.2	c.8559-2A>G<br>c.14243C>T	.<br>p.(Ser4748Phe)	34<br>28.5	0.994<br>0.995	8/251134<br>1/251252	P<br>LP	Infer by gnomAD[c]
GH_0064	*PDE6B*	NM_000283.3	c.1604T>A<br>c.2492C>T	p.(Ile535Asn)<br>p.(Ala831Val)	25.8<br>1.449	0.990<br>0.220	1/250482<br>9/249824	LP<br>LP	Confirmed
GH_0065	*USH2A*	NM_206933.2	c.2802T>G<br>c.14243C>T	p.(Cys934Trp)<br>p.(Ser4748Phe)	25.5<br>28.8	0.974<br>0.995	57/284482<br>1/251252	P<br>LP	Confirmed
GH_0066	*SPATA7*	NM_018418.4	c.388C>T<br>c.657del	p.(Gln130*)<br>p.(Ala220Hisfs*26)	34<br>24.4	0.273<br>0.950	2/249908<br>.	LP<br>P	NC
GH_0068	*RPGR*	NM_001034853.1	c.2961_2968dup	p.(Gly990Glufs*102)	23.5	0.021	.	P	No need
GH_0069	*ABCA4*	NM_000350.2	c.4748T>C<br>c.4762A>T	p.(Leu1583Pro)<br>p.(Asn1588Tyr)	25.6<br>32	0.994<br>0.991	2/251476<br>.	LP<br>LP	IGV confirmed
GH_0070	*NIPBL*	NM_015384.4	c.1237C>T	p.(Pro413Ser)	19.49	0.920	1/250274	LP	No need
GH_0071	*GUCY2D*	NM_000180.3	c.2984G>A<br>c.3044-1G>C	p.(Arg995Gln)<br>.	31<br>33	0.992<br>0.993	1/244998<br>2/243406	LP<br>P	IGV confirmed
GH_0072	*RP1*	NM_006269.1	c.4052_4053ins328<br>c.4582_4585del	p.(Tyr1352Alafs*9)<br>p.(Ile1528Valfs*10)	NA<br>25.7	NA<br>0.913	.<br>4/281542	P<br>P	Confirmed
GS_0073	*RP1*	NM_006269.1	c.2238_2239del	p.(Ser747*)	25.6	0.948	.	P	No need
GH_0074	*PDE6C*	NM_006204.3	c.480G>T<br>exon 1 deletion	p.(Lys160Asn)<br>.	33<br>NA	0.995<br>NA	1/245444<br>.	LP<br>P	Confirmed
GH_0075	*HPS5*	NM_181507.1	c.646_647del<br>c.1134T>A	p.(Asp216Trpfs*28)<br>p.(Cys378*)	32<br>36	0.988<br>0.964	.<br>.	P<br>P	Confirmed

© 2024 Surl D et al. *JAMA Network Open.*

GH_0077	*RPGR*	NM_001034853.1	c.2937_2938del	p.(Glu980Glyfs*98)	24	0.100	.	P	No need
GH_0080	*CNGA3*	NM_001298.2	c.829C>T<br>c.829C>T	p.(Arg277Cys)<br>p.(Arg277Cys)	31	0.936	24/251350	LP	No need
GH_0081	*RP1*	NM_006269.1	c.4196del<br>c.5797C>T	p.(Cys1399Leufs*5)<br>p.(Arg1933*)	22.1<br>38	0.826<br>0.594	1/250622<br>49/281934	P<br>LP	Infer by gnomAD[c]
GS_0083	*USH2A*	NM_206933.2	c.2802T>G<br>exon 47 deletion	p.(Cys934Trp)<br>.	25.5<br>NA	0.974<br>NA	57/282482<br>.	LP<br>P	Confirmed
GH_0085	*COL11A1*	NM_080629.2	c.3852+2dup	.	23.6	0.969	1/251352	P	No need
GH_0086	*CHM*	NM_000390.3	c.715C>T	p.(Arg239*)	36	0.968	1/182788	P	No need
GH_0087	*SOX10*	NM_006941.3	exon 4 deletion	.	NA	NA	.	P	No need
GH_0088[b]	*SLC38A8*	NM_001080442.1	c.682G>A<br>c.695A>G	p.(Gly228Arg)<br>p.(His232Arg)	31<br>23.5	0.988<br>0.948	7/282722<br>1/234770	LP<br>LP	Confirmed
	FRMD7	NM_194277.2	c.875T>C	p.(Leu292Pro)	27.5	0.991	4/183318	LP	No need
GH_0090	*EYS*	NM_001142800.1	c.525_527del<br>c.6557G>A	p.(Glu176del)<br>p.(Gly2186Glu)	17.98<br>22.8	0.990<br>0.815	27/282652<br>6/149126	P<br>LP	Confirmed
GH_0092	*SNRNP200*	NM_014014.4	c.2042G>A	p.(Arg681His)	32	0.985	.	LP	No need
GH_0094	*RAB28*	NM_001017979.2	c.68C>T<br>c.68C>T	p.(Ser23Phe)<br>p.(Ser23Phe)	26	0.666	7/219238	LP	No need
GH_0095	*CACNA1F*	NM_005183.2	c.5479C>T	p.(Arg1827*)	36	0.202	1/183192	P	No need
GH_0096	*PRPH2*	NM_000322.4	c.657_662del	p.(Arg220_Pro221del)	22.3	0.995	.	LP	No need
GH_0101	*EYS*	NM_001142800.1	c.7492G>C<br>c.9248G>A	p.(Ala2498Pro)<br>p.(Gly3083Asp)	23<br>23.5	0.913<br>0.982	7/156982<br>.	LP<br>LP	NC
GH_0102	*CEP290*	NM_025114.3	c.635dup<br>c.4661_4663del	p.(Asn212Lysfs*3)<br>p.(Glu1554del)	27.2<br>21.6	0.894<br>0.959	24/235106<br>7/280272	P<br>LP	Confirmed
GH_0103	*NR2F1*	NM_005654.5	c.51_69dup	p.(Asn24Glyfs*379)	24	0.470	.	LP	No need
GH_0104	*RAB28*	NM_001017979.2	c.68C>T<br>c.68C>T	p.(Ser23Phe)<br>p.(Ser23Phe)	26	0.666	7/219238	P	No need
GH_0106	*ABCA4*	NM_000350.2	c.1522C>T<br>c.1906C>A<br>c.768G>A	p.(Arg508Cys)<br>p.(Gln636Lys)<br>p.(Val256=)	24.3<br>25.6<br>23	0.953<br>0.978<br>0.967	47/282456<br>.<br>.	LP<br>LP<br>LP	NC

© 2024 Surl D et al. *JAMA Network Open.*

GH_0107	*RPE65*	NM_000329.2	c.335G>A<br>c.361dup	p.(Cys112Tyr)<br>p.(Ser121Phefs*10)	29.5<br>32	0.980<br>0.945	1/251396<br>.	LP<br>P	IGV confirmed
GH_0108	*USH2A*	NM_206933.2	c.251G>A<br>c.14287G>A	p.(Cys84Tyr)<br>p.(Gly4763Arg)	23.2<br>25.7	0.914<br>0.997	.<br>.	LP<br>LP	NC
GH_0109	*FGFR1*	NM_023110.2	c.1309A>G	p.(Met437Val)	19.42	0.866	.	VUS	No need
GH_0111	*EYS*	NM_001142800.1	c.6557G>A<br>c.7492G>C	p.(Gly2186Glu)<br>p.(Ala2498Pro)	22.8<br>23	0.815<br>0.913	6/149126<br>7/156982	LP<br>LP	Infer by gnomAD[c]
GH_0112	*PAX6*	NM_000280.4	c.1183+2T>A	.	33	0.977	.	P	No need
GH_0113	*PDE6B*	NM_000283.3	c.1669C>T<br>c.1669C>T	p.(His557Tyr)<br>p.(His557Tyr)	25.6	0.961	6/201374	P	No need
GH_0118	*EYS*	NM_001142800.1	c.4957dup<br>c.4957dup	p.(Ser1653Lysfs*2)<br>p.(Ser1653Lysfs*2)	35	0.853	1/151914	P	No need
GH_0119	*SPATA7*	NM_018418.4	c.20_23del<br>c.388C>T	p.(Val7Glufs*19)<br>p.(Gln130*)	33<br>34	0.605<br>0.273	135/282228<br>2/249908	LP<br>P	Infer by gnomAD[c]
GH_0120	*CNGA3*	NM_001298.2	c.553C>G<br>c.847C>T	p.(Leu185Val)<br>p.(Arg283Trp)	20.5<br>26.5	0.190<br>0.288	1/251394<br>25/251318	LP<br>P	Confirmed
GH_0125	*ABCA4*	NM_000350.2	c.880C>T<br>c.4383G>A	p.(Gln294*)<br>p.(Trp1461*)	36<br>50	0.973<br>0.997	.<br>.	P<br>P	NC
GH_0126	-	-	chr 16p11.2 deletion	.	NA	NA	.	P	No need
GH_0129	*ABCA4*	NM_000350.2	c.454C>T<br>c.880C>T	p.(Arg152*)<br>p.(Gln294*)	40<br>36	0.965<br>0.973	4/251172<br>.	P<br>P	Confirmed
GH_0131	*PROM1*	NM_006017.2	c.1192C>T<br>c.1877_1878del	p.(Gln398*)<br>p.(Ile626Argfs*6)	31<br>32	0.016<br>0.693	.<br>1/249108	P<br>P	NC
GH_0132	*RP2*	NM_006915.2	exon 1 deletion	.	NA	NA	.	P	No need
GS_0133	*EYS*	NM_001142800.1	c.4957dup<br>c.8805C>A	p.(Ser1653Lysfs*2)<br>p.(Tyr2935*)	35<br>35	0.853<br>0.481	1/151914<br>7/188242	P<br>P	Infer by gnomAD[c]
GH_0135	*PAX2*	NM_000278.3	c.95C>G	p.(Pro32Arg)	29.2	0.989	.	LP	No need
GH_0136	*PAX6*	NM_000280.4	chr 11p13 deletion	.	NA	NA	.	P	No need
GH_0138	*ABCA4*	NM_000350.2	c.2948C>T<br>c.4762A>T	p.(Thr983Ile)<br>p.(Asn1588Tyr)	24.3<br>32	0.972<br>0.991	1/251490<br>.	LP<br>LP	NC

© 2024 Surl D et al. *JAMA Network Open.*

GH_0139	*MYO7A*	NM_000260.3	c.799A>T<br>c.4568+1G>A	p.(Lys267*)<br>.	40<br>33	0.994<br>0.975	.<br>.	P<br>P	NC
GH_0140	*USH2A*	NM_206933.2	c.8254G>A<br>c.13351_13353del	p.(Gly2752Arg)<br>p.(Thr4451del)	32<br>14.55	0.944<br>0.988	6/247260<br>.	LP<br>LP	NC
GH_0142	*SLC45A2*	NM_016180.4	c.217G>T<br>c.469G>A	p.(Val73Leu)<br>p.(Asp157Asn)	26.6<br>29.6	0.993<br>0.986	.<br>1/251482	LP<br>LP	Confirmed
GS_0144	*CYP4V2*	NM_207352.3	c.992A>C<br>c.1020G>A	p.(His331Pro)<br>p.(Trp340*)	25.6<br>41	0.997<br>0.967	19/251496<br>1/251496	LP<br>P	IGV confirmed
GH_0146	*MT-ATP6*	NC_012920.1	m.8993T>G	p.(Leu156Arg)	24	NA	.	P	No need
GH_0147	*FZD4*	NM_012193.3	c.160C>T	p.(Gln54*)	37	0.913	.	P	No need
GS_0150	*RS1*	NM_000330.3	c.306_308dup	p.(Leu103dup)	21.9	0.989	.	LP	No need
GS_0151	*PRPH2*	NM_000322.4	c.499G>A	p.(Gly167Ser)	28.8	0.983	.	LP	No need
GS_0153	*CRX*	NM_000554.4	c.121C>T	p.(Arg41Trp)	25.6	0.791	2/251204	LP	No need
GJ_0154	*EYS*	NM_001142800.1	c.4957dup<br>c.8868C>A	p.(Ser1653Lysfs*2)<br>p.(Tyr2935*)	35<br>35	0.853<br>0.481	1/151914<br>7/188242	P<br>P	Infer by gnomAD[c]
GS_0155	*USH2A*	NM_206933.2	c.1724G>A<br>c.8885T>G	p.(Cys575Tyr)<br>p.(Leu2962Arg)	24.1<br>25	0.974<br>0.985	1/251322<br>.	LP<br>LP	NC
GS_0156	*RP1*	NM_006269.2	c.6181del<br>c.6181del	p.(Ile2061Serfs*12)<br>p.(Ile2061Serfs*12)	25.5	0.936	8/282624	P	No need
GS_0157	*USH2A*	NM_206933.2	c.1190T>A<br>c.1804G>A	p.(Ile397Lys)<br>p.(Gly602Arg)	25.6<br>26.8	0.996<br>0.980	.<br>.	LP<br>LP	NC
GS_0158	*RHO*	NM_000539.3	c.541G>A	p.(Glu181Lys)	26	0.988	.	LP	No need
GJ_0159	*RHO*	NM_000539.3	c.541G>A	p.(Glu181Lys)	26	0.988	.	LP	No need
GH_0160	*RP2*	NM_006915.2	c.352C>T	p.(Arg118Cys)	32	0.979	.	LP	No need
GH_0163	*LRP5*	NM_002335.2	c.4488+1G>A	.	35	0.994	.	LP	No need
GH_0166	*GPR143*	NM_000273.2	c.14G>C	p.(Arg5Pro)	27.7	0.921	.	LP	No need
GH_0167	*ABCA4*	NM_000350.2	c.2424C>G<br>c.6389T>A	p.(Tyr808*)<br>p.(Met2130Lys)	36<br>29.3	0.950<br>0.970	.<br>.	P<br>LP	Confirmed
GH_0169	*GPR143*	NM_000273.2	c.711del	p.(Met238Trpfs*4)	23.3	0.957	.	P	No need
GH_0171	*GPR143*	NM_000273.2	c.248T>C	p.(Leu83Pro)	25.7	0.565	.	LP	No need

© 2024 Surl D et al. *JAMA Network Open.*

GH_0172	*ABCA4*	NM_000350.2	c.1760+2T>G<br>c.5714+5G>A	.<br>.	25.7<br>22.1	0.950<br>0.979	.<br>84/282690	P<br>LP	NC
GH_0174	*CNGB1*	NM_001297.4	c.217+5G>C<br>c.2653_2654insGA	.<br>p.(Ala885Glyfs*18)	23.2<br>33	0.972<br>0.994	13/280826<br>.	LP<br>P	NC
GH_0178	*PDE6C*	NM_006204.3	c.1646T>C<br>c.1766A>G	p.(Met549Thr)<br>p.(Asp589Gly)	22.5<br>24.6	0.864<br>0.983	2/251386<br>.	LP<br>LP	Confirmed
GH_0179	*PAX6*	NM_000280.4	c.10+5G>C	.	27	0.926	.	LP	No need
GH_0181	*POC1B*	NM_172240.3	c.880G>T<br>c.1336_1337del	p.(Val294Phe)<br>p.(Val446Phefs*13)	32<br>34	0.937<br>0.957	6/244908<br>1/240700	LP<br>P	Infer by gnomAD[c]
GH_0182	*PTPN23*	NM_015466.3	c.3768del<br>c.4886C>G	p.(Pro1258Argfs*2)<br>p.(Pro1629Arg)	31<br>25.5	0.990<br>0.990	.<br>.	P<br>LP	Confirmed
GH_0184	*MT-ATP6*	NC_012920.1	m.8993T>G	p.(Leu156Arg)	24	NA	.	P	No need
GH_0185[b]	*NMNAT1*	NM_022787.3	c.29T>G<br>c.710G>A	p.(Val10Gly)<br>p.(Arg237His)	25.2<br>23.5	0.995<br>0.984	.<br>13/282770	LP<br>LP	Confirmed
	RHO	NM_000539.3	c.302G>T	p.(Gly101Val)	25.2	0.995	.	LP	No need
GH_0186	*USH2A*	NM_206933.4	c.251G>A<br>c.2802T>G	p.(Cys84Tyr)<br>p.(Cys934Trp)	23.2<br>25.5	0.914<br>0.974	.<br>57/282482	LP<br>P	NC
GH_0188	*USH2A*	NM_206933.4	c.12730A>C<br>c.14792-2A>G	p.(Asn4244His)<br>.	26<br>34	0.997<br>0.994	.<br>2/251336	LP<br>P	NC
GH_0189	*OPA1*	NM_015560.2	c.1674_1676dup	p.(Ser559dup)	18.8	0.997	.	LP	No need
GJ_0161	*ARSG*	NM_001267727.2	c.982+1G>C<br>exon 5 deletion	.<br>.	25.8<br>NA	0.984<br>NA	1/250984	P<br>LP	NC
GS_0190	*PDE6A*	NM_000440.3	c.948C>G<br>c.1283G>A	p.(Tyr316*)<br>p.(Gly428Asp)	39<br>29.8	0.928<br>0.981	1/249544<br>2/250390	LP<br>LP	Infer by gnomAD[c]
GS_0191	*RP1*	NM_006269.1	c.4052_4053ins328<br>c.5797C>T	p.(Tyr1352Alafs*9)<br>p.(Arg1933*)	NA<br>38	NA<br>0.594	.<br>49/281934	P<br>LP	NC
GS_0192	*RPGR*	NM_001034853.2	c.2236_2237del	p.(Glu746Argfs*23)	21.7	0.021	.	P	No need
GS_0193	*BEST1*	NM_004183.4	c.113T>G<br>c.140G>A	p.(Ile38Ser)<br>p.(Arg47His)	21.3<br>14.55	0.977<br>0.988	.<br>4/251182	LP<br>LP	IGV confirmed
GH_0195[b]	*BEST1*	NM_004183.4	c.632T>C<br>c.809A>G	p.(Leu211Pro)<br>p.(Asp270Gly)	26.2<br>26.4	0.995<br>0.981	.<br>.	LP<br>LP	Confirmed

© 2024 Surl D et al. *JAMA Network Open.*

	PTPN11	NM_002834.5	c.417G>C	p.(Glu139Asp)	25	0.961	.	LP	No need
GH_0198	*TSPAN12*	NM_012338.4	c.194C>T	p.(Pro65Leu)	22.9	0.944	2/251238	LP	No need
GH_0199	*ABCA4*	NM_000350.2	c.1357-1G>C<br>c.2948C>T	.<br>p.(Thr983Ile)	33<br>24.3	0.996<br>0.972	.<br>1/251490	P<br>LP	NC
GH_0201	*MT-ND4*	NC_012920.1	m.11778G>A	p.(Arg340His)	24.4	NA	11/56423	P	No need
BDC_0001	*ABCA4*	NM_000350.2	c.1921T>G<br>c.6289C>T	p.(Cys641Gly)<br>p.(Pro2097Ser)	25<br>29.7	0.968<br>0.993	.<br>.	LP<br>LP	NC
BDC_0002	*RP1*	NM_006269.1	c.4196del<br>c.5797C>T	p.(Cys1399Leufs*5)<br>p.(Arg1933*)	22.1<br>38	0.826<br>0.594	1/250622<br>49/281934	P<br>LP	Infer by gnomAD[c]
BDC_0003	*RP1*	NM_006269.1	c.5805dup<br>c.6181del	p.(Ala1936Cysfs*7)<br>p.(Ile2061Serfs*12)	34<br>25.5	0.963<br>0.936	1/250638<br>8/282624	P<br>P	Infer by gnomAD[c]
BDC_0005	*EYS*	NM_001142800.1	c.813_816dup<br>c.6557G>A	p.(Ile273Glnfs*6)<br>p.(Gly2186Glu)	16.71<br>22.8	0.768<br>0.815	.<br>6/149126	LP<br>LP	NC
BDC_0006	*ABCA4*	NM_000350.2	c.1357-1G>C<br>c.4383G>A	.<br>p.(Trp1461*)	33<br>50	0.996<br>0.997	.<br>.	P<br>P	NC
BDC_0007	*ABCA4*	NM_000350.2	c.1357-1G>C<br>c.4383G>A	.<br>p.(Trp1461*)	33<br>50	0.996<br>0.997	.<br>.	P<br>P	NC
BDC_0008	*RPGR*	NM_001034853.2	c.2293G>T	p.(Glu765*)	28.9	0.069	.	P	No need
BDC_0009	*ABCA4*	NM_000350.2	c.1760+2T>G<br>c.6119G>A	.<br>p.(Arg2040Gln)	34<br>25.1	0.950<br>0.988	.<br>45/152140	P<br>LP	NC
BDC_0010	*ABCA4*	NM_000350.2	c.1357-1G>C<br>c.4748T>C	.<br>p.(Leu1583Pro)	33<br>25.6	0.996<br>0.994	.<br>2/251476	P<br>LP	NC
BDC_0012	*ABHD12*	NM_001042472.2	c.897G>A<br>exon 10 deletion	p.(Trp299*)<br>.	41<br>NA	0.977<br>NA	.<br>.	P<br>P	IGV confirmed

Abbreviations: ACMG, American College of Medical Genetics; LP, likely pathogenic; P, pathogenic; VUS, variant of uncertain significance.
[a]The minor allele frequency was from gnomAD v.2.1.1 (hg19).
[b]These patients had pathogenic variants with other genes, possibly dual molecular diagnosis (autosomal recessive + autosomal dominant or autosomal recessive + X-linked).
[c]The phase of two variants were inferred using gnomAD variant co-occurrence.[8]

© 2024 Surl D et al. *JAMA Network Open.*

eTable 3. Novel disease-causing variants, *in silico* predictions, and minor allele frequencies.

Gene	Diagnosis	Transcript	Nucleotide change	Amino acid change	Domain[a]	CADD	FATHMM	gnomAD[b]
ABCA4	Stargardt	NM_000350.2	c.664dup	p.(Ala222Glyfs*58)	Rim ABC transporter	23.3	0.955	Not found
		NM_000350.2	c.1357-1G>C	.	-	33	0.996	Not found
		NM_000350.2	c.5312G>T	p.(Gly1771Val)	Rim ABC transporter	35	0.987	Not found
ABHD12	RP	NM_001042472.2	c.897G>A	p.(Trp299*)	-	41	0.977	Not found
		NM_001042472.2	exon 10 deletion	.	-	NA	NA	Not found
ARSG	RP	NM_001267727.2	Exon 5 deletion	.	Sulfatase, N-terminal	NA	NA	Not found
BBS1	BBS	NM_024649.5	c.159G>C	p.(Lys53Asn)	-	36	0.967	Not found
		NM_024649.5	exon 10-12 deletion	.	Quinonprotein alcohol dehydrogenase-like superfamily	NA	NA	Not found
BBS9	BBS	NM_198428.2	c.1016+4del	.	-	24.8	0.984	Not found
		NM_198428.2	exon 14-19 deletion	.	-	NA	NA	Not found
BEST1	Bestro-phinopathy	NM_004183.4	c.632T>C[c]	p.(Leu211Pro)	Bestrophin/UPF0187	26.2	0.995	Not found
		NM_004183.4	c.809A>G	p.(Asp270Gly)	Bestrophin/UPF0187	26.4	0.981	Not found
CERKL	RP	NM_001030311.3	c.1283_1286dup	p.(Ile430Glnfs*14)	-	35	0.973	Not found
CHM	Choroideremia	NM_000390.4	c.403_404del	p.(Asp135Phefs*9)	-	22.2	0.755	Not found
CNGB1	RP	NM_001297.4	c.2154C>T	p.(Gly718=)	-	15.9	0.088	12/248428
		NM_001297.4	c.2653_2654insGA	p.(Ala885Glyfs*18)	-	33	0.994	Not found
CRB1	LCA/EOSRD	NM_201253.3	c.1577G>C	p.(Arg526Pro)	-	23	0.622	Not found
EYS	RP	NM_001142800.1	c.813_816dup	p.(Ile273Glnfs*6)	Epidermal growth factor-like domain	16.71	0.768	Not found
		NM_001142800.1	c.2471G>A	p.(Cys824Tyr)	Epidermal growth factor-like domain	18.93	0.876	Not found
		NM_001142800.1	c.6395T>C	p.(Leu2132Pro)	Epidermal growth factor-like domain	26	0.895	Not found
		NM_001142800.1	c.8963C>T	p.(Thr2988Ile)	Laminin G domain	23.8	0.966	Not found
		NM_001142800.1	c.9340G>C	p.(Asp3114His)	Laminin G domain	24.4	0.975	Not found
		NM_001142800.1	exon 4 deletion	.	Epidermal growth factor-like domain	NA	NA	Not found
		NM_001142800.1	exon 4-5 deletion	.	Epidermal growth factor-like domain	NA	NA	Not found
GPR143	OA	NM_000273.2	c.711del	p.(Met238Trpfs*4)	Ocular albinism protein, type 1	23.3	0.957	Not found
		NM_000273.2	c.925del	p.(Ala309Profs*24)	Ocular albinism protein, type 1	34	0.929	Not found

© 2024 Surl D et al. *JAMA Network Open.*

GUCY2D	LCA/EOSRD	NM_000180.3	c.3044-1G>C	.	-	33	0.993	2/243406
HPS5	OCA	NM_181507.1	c.646_647del	p.(Asp216Trpfs*28)	BLOC-2 complex, Hps5 subunit	32	0.988	Not found
		NM_181507.1	c.1134T>A	p.(Cys378*)	BLOC-2 complex, Hps5 subunit	36	0.964	Not found
IMPDH1	RP	NM_000883.3	c.147-2A>G	.	-	24.5	0.389	Not found
IMPG1	RP	NM_001563.4	c.1896T>G	p.(Ser632Arg)	SEA domain	23.7	0.882	Not found
KIZ	RP	NM_018474.6	c.62_65del	p.(Gly21Aspfs*33)	-	23.3	0.535	Not found
LRP5	FEVR	NM_002335.2	c.4488+1G>A	.	-	35	0.994	Not found
MAK	RP	NM_005906.5	c.502C>G	p.(Pro168Ala)	Protein kinase domain	25.2	0.973	1/251388
MYO7A	Usher	NM_000260.3	c.799A>T	p.(Lys267*)	Myosin head, motor domain	40	0.994	Not found
NBAS	SOPH	NM_015909.3	c.3494del[c]	p.(Val1165Serfs*31)	Secretory pathway sec39	34	0.934	Not found
		NM_015909.3	c.5740C>T[c]	p.(Arg1914Cys)	-	24.9	0.942	Not found
NIPBL	CdLS	NM_133433.4	c.1237C>T	p.(Pro413Ser)	-	19.49	0.920	1/250274
NMNAT1	LCA/EOSRD	NM_022787.3	c.29T>G	p.(Val10Gly)	-	25.2	0.995	Not found
		NM_022787.3	exon 2 deletion[c]	.	Cytidylyltransferase	NA	NA	Not found
NR2F1	BBSOAS	NM_005654.5	c.51_69dup[c]	p.(Asn24Glyfs*379)	-	24	0.470	Not found
OPA1	DOA	NM_015560.2	c.1674_1676dup	p.(Ser559dup)	P-loop containing nucleoside triphosphate hydrolase	18.8	0.997	Not found
PAX2	Papillorenal syndrome	NM_000278.3	c.95C>G	p.(Pro32Arg)	Paired domain	29.2	0.989	Not found
PAX6	Aniridia	NM_000280.4	c.1183+2T>A	.	-	33	0.977	Not found
PDE6B	RP	NM_000283.3	c.2068C>T	p.(Gln690*)	3'5'-cyclic nucleotide phosphodiesterase, catalytic domain	36	0.427	Not found
PDE6C	ACHM	NM_006204.3	c.1766A>G[c]	p.(Asp589Gly)	3'5'-cyclic nucleotide phosphodiesterase, catalytic domain	24.6	0.983	Not found
		NM_006204.3	exon 1 deletion[c]	.	-	NA	NA	Not found
POC1B	CRD	NM_172240.3	c.880G>T	p.(Val294Phe)	WD40 repeat	32	0.937	6/244908
PROM1	LCA/EOSRD	NM_006017.2	c.1192C>T	p.(Gln398*)	Prominin	31	0.016	Not found
PRPF31	RP	NM_015629.3	exon 6-14 duplication	.	-	NA	NA	Not found
PTPN23	OA	NM_015466.3	c.3768del[c]	p.(Pro1258Argfs*2)	Protein-tyrosine phosphatase, receptor/non-receptor type	31	0.990	Not found

© 2024 Surl D et al. *JAMA Network Open.*

		NM_015466.3	c.4886C>G[c]	p.(Pro1629Arg)	-	25.5	0.990	Not found
RAB28	CD	NM_001017979.3	c.76-158T>G	.	-	12.13	0.171	Not found
RP1	RP	NM_006269.1	c.289G>T	p.(Glu97*)	Doublecortin domain RP1/RP1L1/DCX	35	0.966	Not found Not found
RP2	RP	NM_006915.2	c.100_102+17del	p.(Lys34del)	Protein XRP2	24.7	0.167	Not found
RPGR	RP	NM_001034853.1	c.310+1G>T	.	-	34	0.996	Not found
		NM_001034853.1	c.2779del	p.(Glu927Lysfs*162)	-	23.4	0.025	Not found
		NM_001034853.1	c.2961_2968dup	p.(Gly990Glufs*102)	-	23.5	0.021	Not found
SLC38A8	Foveal hypoplasia	NM_001080442.2	c.682G>A[c]	p.(Gly228Arg)	Amino acid transporter, transmembrane	31	0.988	7/282722
		NM_001080442.2	c.695A>G[c]	p.(His232Arg)	Amino acid transporter, transmembrane	23.5	0.948	1/234770
SOX10	SWS	NM_006941.3	exon 4 deletion	.	-	NA	NA	Not found
USH2A	RP	NM_206933.2	c.1645-2A>C	.	-	34	0.989	Not found
		NM_206933.2	c.10973T>C	p.(Leu3648Pro)	Fibronectin, type 3	24.5	0.977	Not found
		NM_206933.4	c.12730A>C	p.(Asn4244His)	Fibronectin, type 3	26	0.997	Not found
		NM_206933.2	c.13351_13353del	p.(Thr4451del)	Fibronectin, type 3	14.55	0.988	Not found
		NM_206933.2	exon 47 deletion	.	Fibronectin, type 3	NA	NA	Not found

Abbreviations: ACHM, achromatopsia; BBS, Bardet Biedl syndrome; BBSOAS, Bosch-Boonstra-Schaaf optic atrophy syndrome; CdLS, Cornellia de Lange syndrome; CD, cone dystrophy; CRD, cone rod dystrophy; DOA, dominant optic atrophy; EOSRD, early-onset severe retinal dystrophy; FEVR, familial exudative vitreoretinopathy; LCA, Leber congenital amaurosis; NC, not confirmed; OA, ocular albinism; OCA, oculocutaneous albinism; RP, retinitis pigmentosa; SOPH, short stature optic atrophy Pelger-Huet anomaly; SWS, Shah-Waardenburg syndrome.

[a]The Alamut Visual Plus (Sophia Genetics, France) was used for the annotation of functional domain.

[b]The minor allele frequency was from gnomAD v.2.1.1 (hg19).

[c]Novel variant, but previously reported by our group.

© 2024 Surl D et al. *JAMA Network Open.*

eTable 4. Splicing variants, *in silico* predictions, and its minor allele frequencies.

Gene	Transcript	Variants	CADD	SpliceAI	gnomAD	Novel
ABCA4	NM_000350.2	c.768G>A:p.(Val256=)	23	Don. loss **0.54** (0bp), Don. gain 0.49 (96bp)	Not found	Reported
		c.1357-1G>C	33	Acc. loss **0.98** (-1bp), Acc. gain 0.28 (-23bp)	Not found	Novel
		c.1760+2T>G	34	Don. loss **0.82** (2bp), Don. gain 0.09 (-35bp)	Not found	Reported
		c.5312G>T:p.(Gly1771Val)	35	Don. loss 0.43 (0bp), Don. gain 0.33 (6bp)	Not found	Novel
		c.5714+5G>A	22.1	Don. loss 0.31 (5bp), Don. gain 0.02 (135bp)	84/282690	Reported
ARSG	NM_001267727.2	c.982+1G>C	25.8	Don. loss **0.99** (-1bp), Acc. loss **0.97** (-81bp)	1/250984	
BBS9	NM_198428.2	c.1016+4del	24.8	Don. loss **0.99** (-3bp), Don. gain 0.23 (-23bp)	Not found	Novel
CEP290	NM_025114.3	c.6012-12T>A	23.3	Acc. loss **0.95** (-12bp), Acc. gain 0.34 (45bp)	4/224124	Reported
CNGB1	NM_001297.4	c.217+5G>C	23.2	Don. loss **0.90** (5bp), Don. gain 0.01 (-125bp)	13/280826	Reported
		c.2154C>T:p.(Gly718=)	15.9	Don. loss 0.23 (-12bp), Don. gain **0.98** (2bp)	12/248428	Reported
COL11A1	NM_080629.2	c.3852+2dupT	23.6	Don. loss **0.74** (2bp), Don. gain 0.44 (10bp)	1/251352	Reported
CYP4V2	NM_207352.3	c.675-1G>A	33	Acc. loss **0.89** (1bp), Acc. gain 0.01 (-74bp)	Not found	Reported
		c.802-8_810del17insGC	NA	Acc. loss **0.99** (9bp), Acc. gain 0.40 (-42bp)	Not found	Reported
EYS	NM_001142800.1	c.1185-6T>G	22.5	Acc. loss 0.16 (-6bp)	1/246084	Reported
GUCY2D	NM_000180.3	c.3044-1G>C	33	Acc. loss **0.99** (1bp), Acc. gain 0.03 (26bp)	2/243406	Novel
IMPDH1	NM_000883.3	c.147-2A>G	24.5	Acc. loss 0.25 (-2bp), Acc. gain 0.37 (-8bp)	Not found	Novel
LRP5	NM_002335.2	c.4488+1G>A	35	Don. loss **0.98** (-1bp), Don. gain 0.44 (-11bp)	Not found	Novel
MYO7A	NM_000260.3	c.4568+1G>A	33	Don. loss **0.99** (-1bp), Don. gain **0.67** (-51bp)	Not found	Reported
PAX6	NM_000280.4	c.1183+2T>A	33	Don. loss **0.99** (2bp), Don. gain 0.01 (96bp)	Not found	Novel
PAX6	NM_000280.4	c.10+5G>C	27	Don. loss **0.88** (5bp), Don. gain 0.04 (-248bp)	Not found	Reported
PDE6C	NM_006204.3	c.480G>T:p.(Lys160Asn)	33	Don. loss 0.12 (0bp), Don. gain 0.27 (-99bp)	1/245444	Reported
POC1B	NM_172240.3	c.880G>T:p.(Val294Phe)	32	Acc. loss 0.05 (0bp), Acc. gain **0.56** (-43bp)	6/244908	Novel
RAB28	NM_004249.4	c.76-158T>G	12.13	Acc. gain **0.81** (102bp), Don. gain **0.92** (1bp)	Not found	Novel
RHO	NM_000539.3	c.937-1G>A	34	Acc. loss **1.00** (1bp), Acc. gain **0.50** (255bp)	Not found	Reported
RP2	NM_006915.2	c.100_102+17del	24.7	Don. loss **1.00** (3bp), Don. gain 0.30 (55bp)	Not found	Novel
RPGR	NM_001034853.1	c.310+1G>T	34	Don. loss **0.95** (1bp), Don. gain 0.01 (-295bp)	Not found	Novel
TYR	NM_000372.4	c.1037-7T>A	18.81	Acc. loss 0.29 (7bp), Acc. gain **0.95** (2bp)	242/280938	Reported
USH2A	NM_206933.2	c.486-14G>A	17.49	Acc. loss **0.95** (-14bp), Acc. gain **0.98** (-2bp)	5/250430	Reported
		c.1645-2A>C	34	Acc. loss **0.97** (-2bp), Acc. gain 0.24 (-35bp)	Not found	Novel
		c.4758+3A>G	20.7	Don. loss 0.41 (3bp), Don. gain 0.03 (-104bp)	422/281358	Reported
		c.8559-2A>G	34	Acc. loss **0.99** (-2bp)	8/251134	Reported
		c.11389+3A>T	22.7	Don. loss **0.90** (3bp), Don. gain 0.13 (46bp)	2//250762	Reported
		c.14792-2A>G	34	Acc. loss **0.99** (-2bp), Acc. gain **0.91** (60bp)	2/251336	Reported

Abbreviations: Acc., acceptor; Don., donor.
Red indicates that spliceAI scores are between 0.2 and 0.5 (high recall cutoff). Red bold indicates that spliceAI scores are equal or more than 0.5 (recommended or high precision cutoffs).[9]

© 2024 Surl D et al. *JAMA Network Open.*

eTable 5. Copy number variations and ACMG/ClinGen score of copy number variations in 17 patients.

Diagnosis	Gene	Transcript	Copy number variation	Inheritance	Zygosity	ACMG/ ClinGen score[a]	ACMG	Novel	Algorithms
ACHM	*PDE6C*	NM_006204.3	exon 1 deletion	AR	hetero	0.9	LP	Novel	ED
Aniridia	*PAX6*	NM_000280.4	chr 11p deletion	AD	hetero	1.3	P	Reported	cn.Mops, ED, GATK
Aniridia	*PAX6*	NM_000280.4	chr 11p13 deletion	AD	hetero	1.3	P	Reported	ED, GATK
BBS	*BBS9*	NM_198428.2	exon14-19 deletion	AR	hetero	1.0	P	Novel	ED
BBS	*BBS1*	NM_024649.5	exon10-12 deletion	AR	hetero	1.0	P	Novel	ED
Choroideremia	*CHM*	NM_000390.4	exon 2-8 duplication	XL	hemi	1.0	P	Novel[b]	ED, cn.Mops
LCA/EOSRD	*NMNAT1*	NM_022787.3	exon 2 deletion	AR	hetero	1.3	P	Novel[b]	Not called
RP	*ABHD12*	NM_001042472.2	exon 10 deletion	AR	hetero	0.9	LP	Novel	ED, GATK
RP	*ARSG*	NM_001267727.2	exon 5 deletion	AR	hetero	0.9	LP	Novel	ED
RP	*EYS*	NM_001142800.1	exon 4 deletion	AR	hetero	1.0	P	Novel[b]	ED
RP	*EYS*	NM_001142800.1	exon 4-5 deletion	AR	hetero	1.0	P	Novel	ED
RP	*EYS*	NM_001142800.1	exon 6-8 deletion	AR	hetero	1.0	P	Novel	ED
RP	*PRPF31*	NM_015629.3	exon 6-14 duplication	AD	hetero	0.4	VUS	Novel	GATK
RP	*RP2*	NM_006915.2	exon 1 deletion	XL	hemi	1.0	P	Reported	ED
RP	*USH2A*	NM_206933.2	exon 47 deletion	AR	hetero	1.0	P	Novel	ED
SWS	*SOX10*	NM_006941.3	exon 4 deletion	AD	hetero	1.0	P	Novel	ED
16p11.2 deletion syndrome	NA	NA	chr 16p11.2 deletion	AD	hetero	1.0	P	Reported	ED, GATK

Abbreviations: ACHM, achromatopsia; ACMG, American College of Medical Genetics; AD, autosomal dominant; AR, autosomal recessive; BBS, Bardet-Biedl syndrome; ED, ExomeDepth; EOSRD, early onset severe retinal dystrophy; GATK, GATK gCNV; LCA, Leber congenital amaurosis; LP, likely pathogenic; NA, not applicable; P, pathogenic; RP, retinitis pigmentosa; VUS, variant of uncertain significance; XL, X-linked.

[a]ACMG/ClinGen score interpretation: Pathogenic: 0.99 or more points; Likely pathogenic: 0.90 to 0.98 points; Uncertain significance: 0.89 to –0.89 points; Likely benign: –0.90 to –0.98 points; Benign: –0.99 or fewer points.

[b]Previously reported patients by our group.

© 2024 Surl D et al. *JAMA Network Open.*

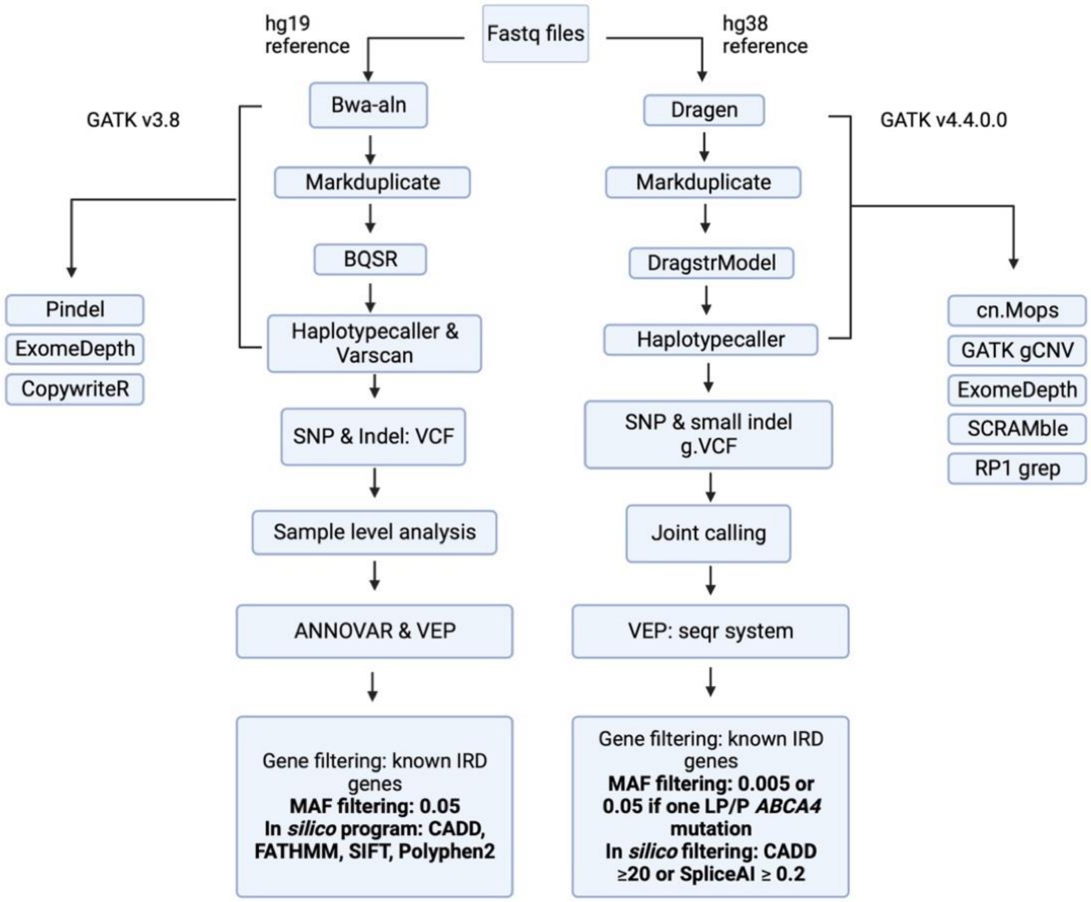

eFigure 1. Schematic Diagram of Next-Generation Sequencing Analysis Workflow

© 2024 Surl D et al. *JAMA Network Open.*

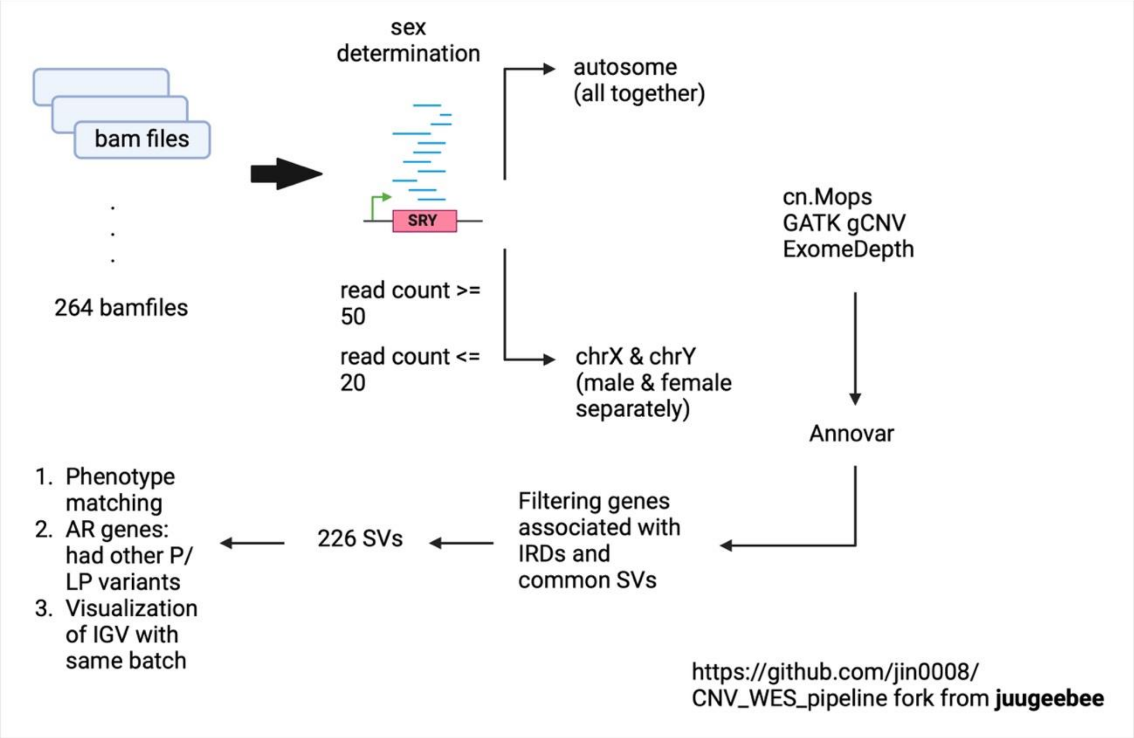

eFigure 2. Workflow of Copy Number Variations Analysis in Exome Sequencing Reanalysis

© 2024 Surl D et al. *JAMA Network Open.*

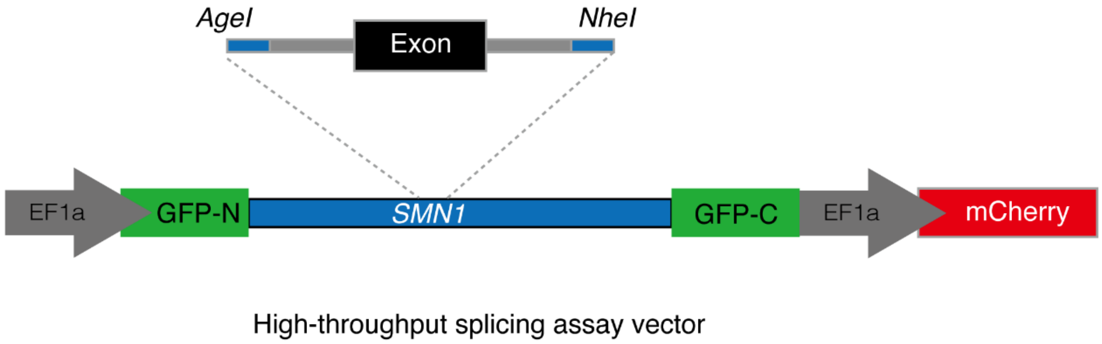

eFigure 3. Schematic presentation of high-throughput splicing assay vector for mini-gene splicing assay

© 2024 Surl D et al. *JAMA Network Open.*

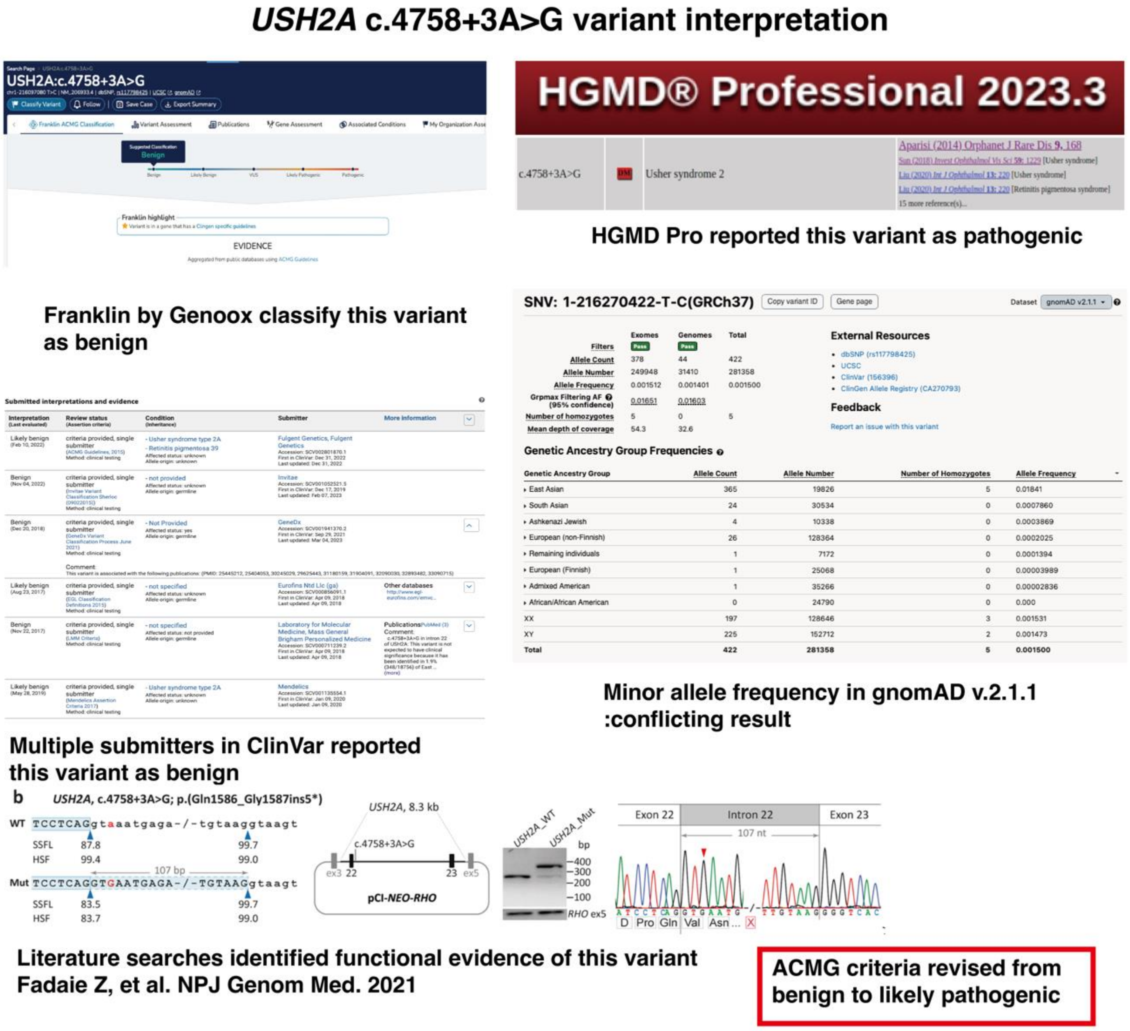

eFigure 4. Manual adjustment American College of Medical Genetics/American Molecular Pathology criteria by searching all available sources such as ClinVar, gnomAD, HGMP Pro, Franklin by Genoox, and literatures.[10]

© 2024 Surl D et al. *JAMA Network Open.*

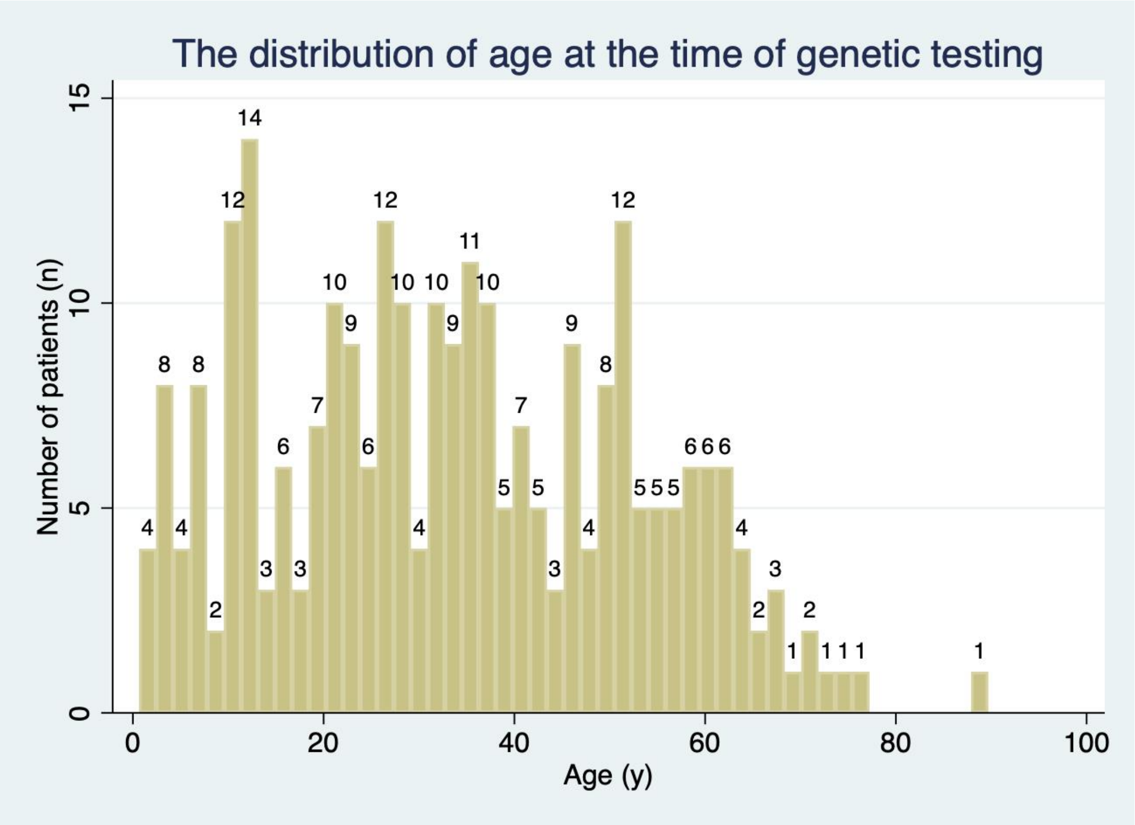

eFigure 5. The Distribution of Age at the time of Genetic Testing

© 2024 Surl D et al. *JAMA Network Open.*

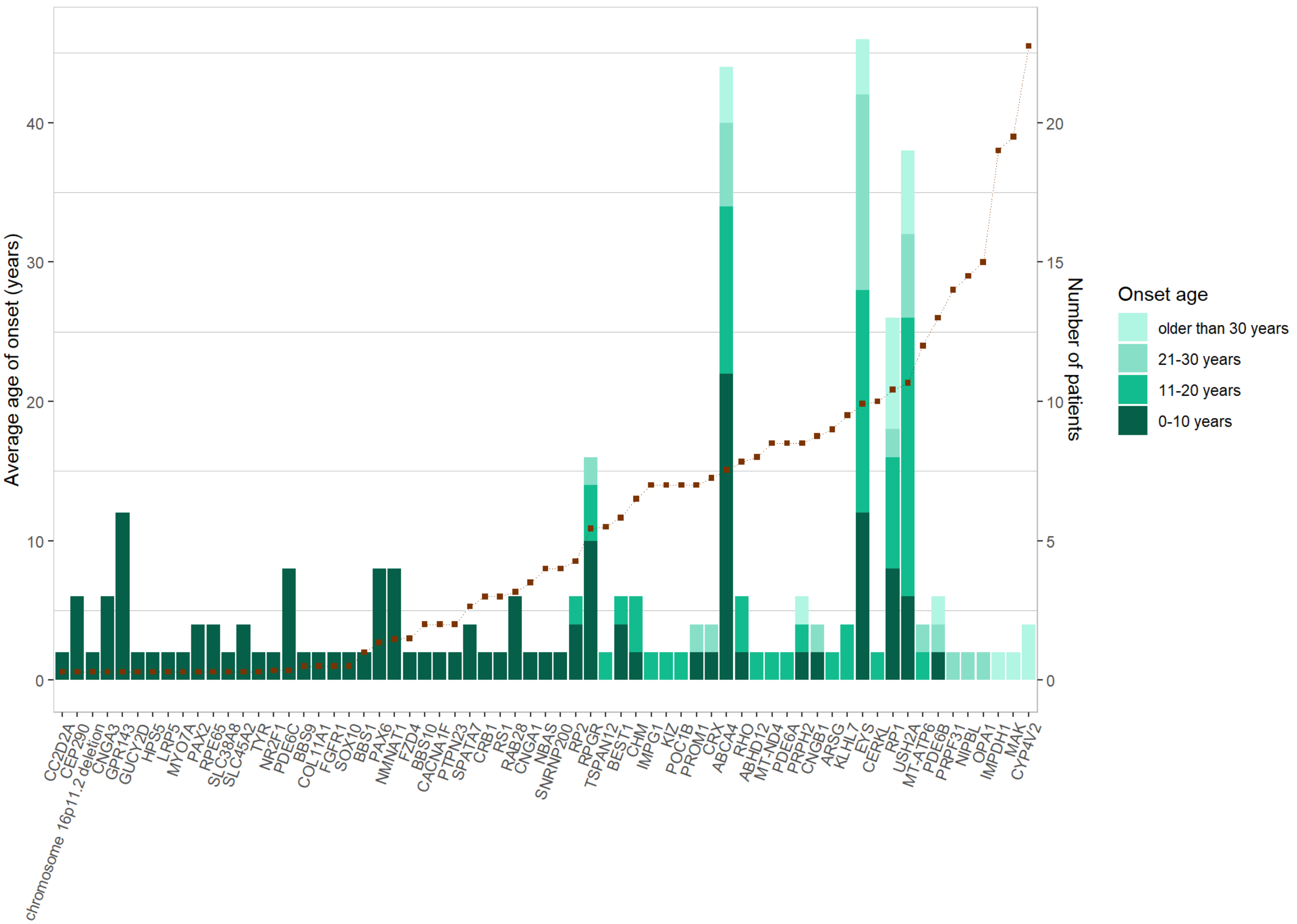

eFigure 6. Distribution of age of onset and total number of cases according to causative genes

© 2024 Surl D et al. *JAMA Network Open.*

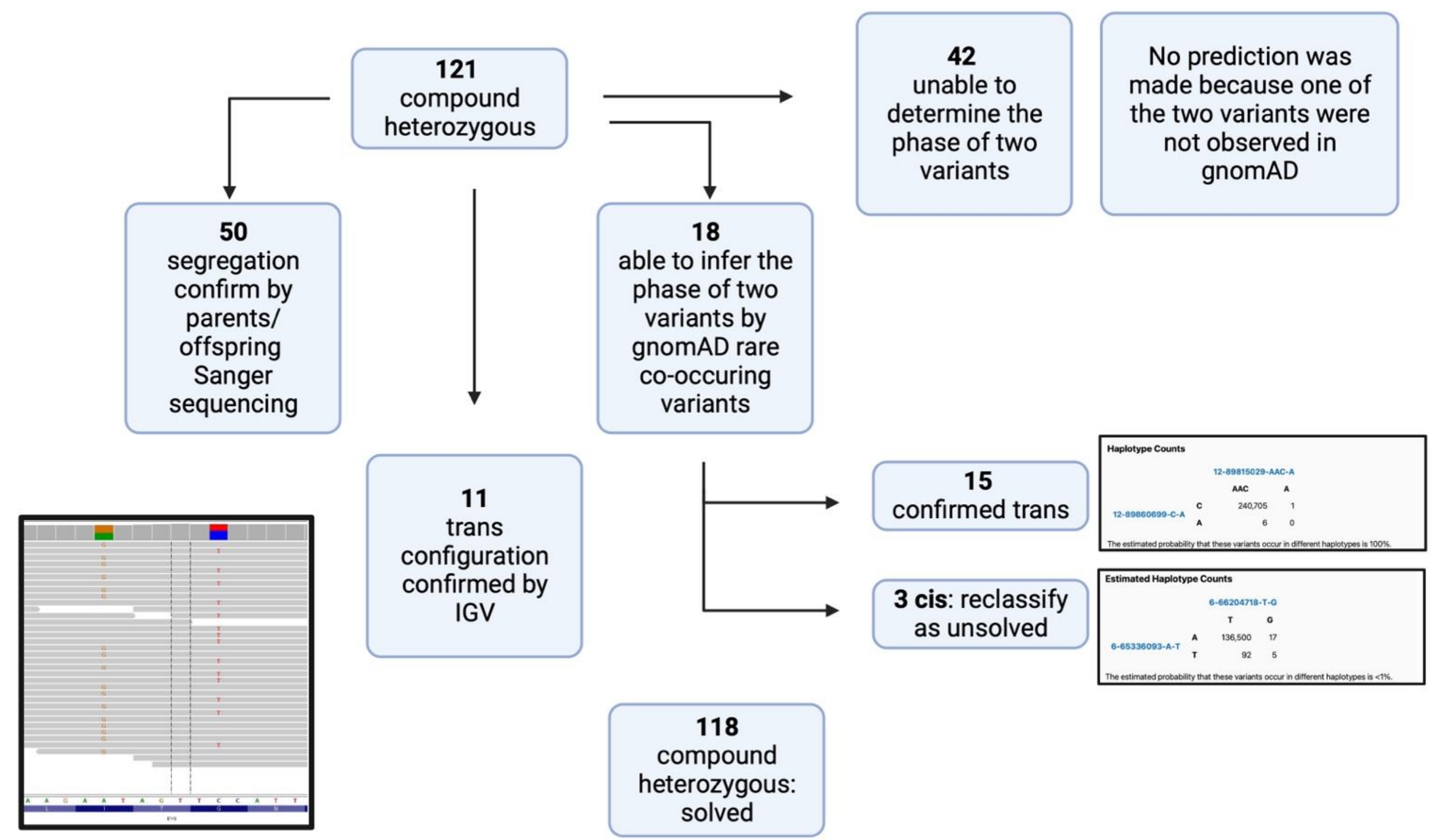

eFigure 7. The segregation analysis in patients with compound heterozygous variants. Among 121 cases with compound heterozygous variants, segregation analysis was done in 50 patients, trans configuration of two variants were confirmed by next generation sequencing reads visualized in the integrated genomics viewer in 11 patients, and the phase of two variants was inferred by gnomAD variant co-occurrence in 15 patients. Three patients were re-classified as unsolved after inferring the phase of two variants.

© 2024 Surl D et al. *JAMA Network Open.*

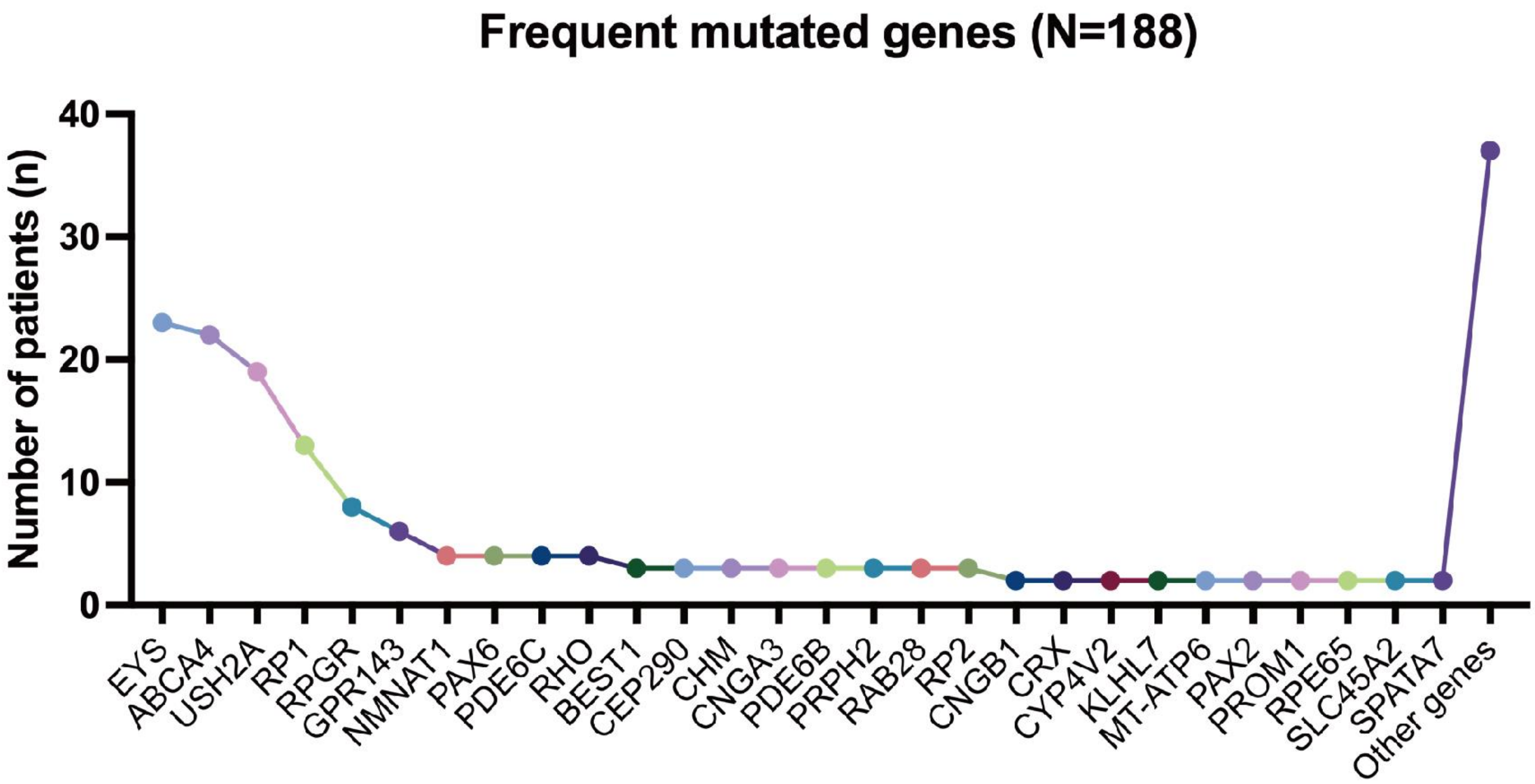

eFigure 8. Frequent mutated genes in this cohort

© 2024 Surl D et al. *JAMA Network Open.*

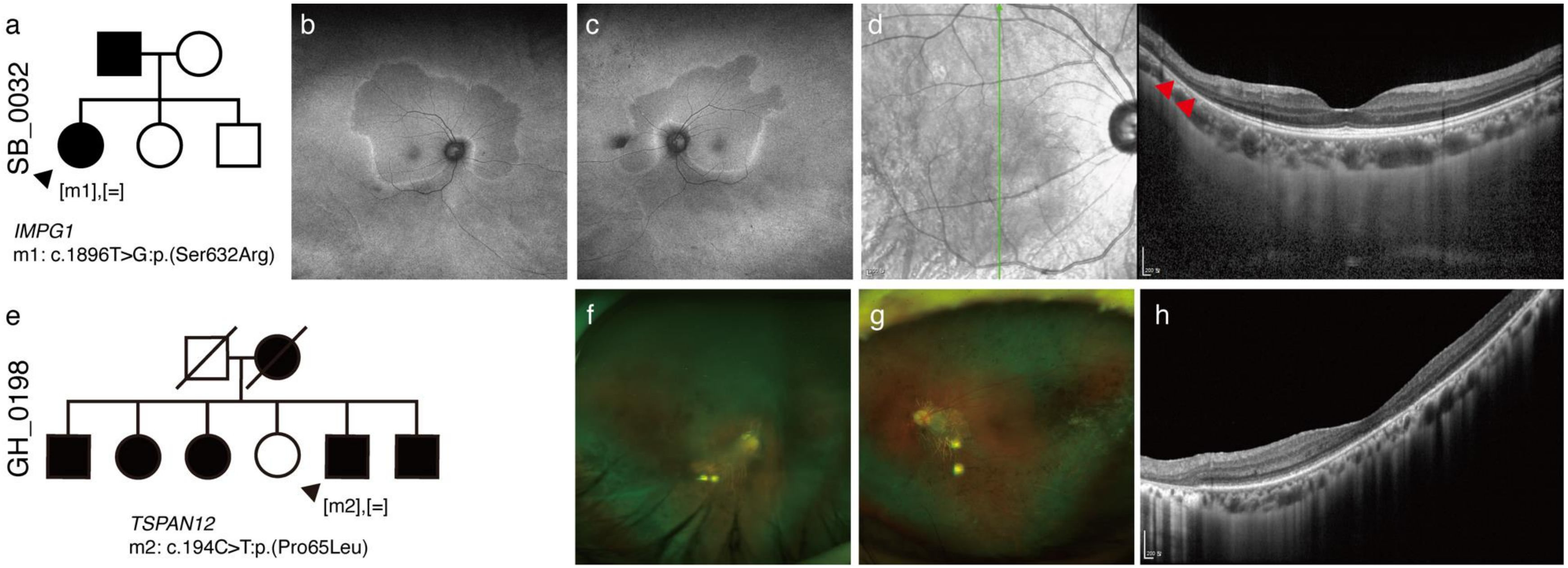

eFigure 9. New molecular diagnoses were made based on update of clinical diagnosis. (a-d) Pathogenic variants in the *IMPG1* gene have previously been described in patients with vitelliform macular dystrophy. However, Olivier el al. reported that variants in *IMPG1* causes autosomal dominant and recessive retinitis pigmentosa (RP) with incomplete penetrance.[11] (e-h) A 47 year-old male who was initially diagnosed with RP came to our clinic for genetic testing. Initial exome sequencing (ES) analysis failed to identify pathogenic variant in this patient. Wide fundus photography showed dragging of retinal vessel as well as degeneration of peripheral retina. ES analysis revealed *TSPAN12* c.194C>T:p.(Pro65Leu) variant, which was matched to patient's phenotype.

© 2024 Surl D et al. *JAMA Network Open.*

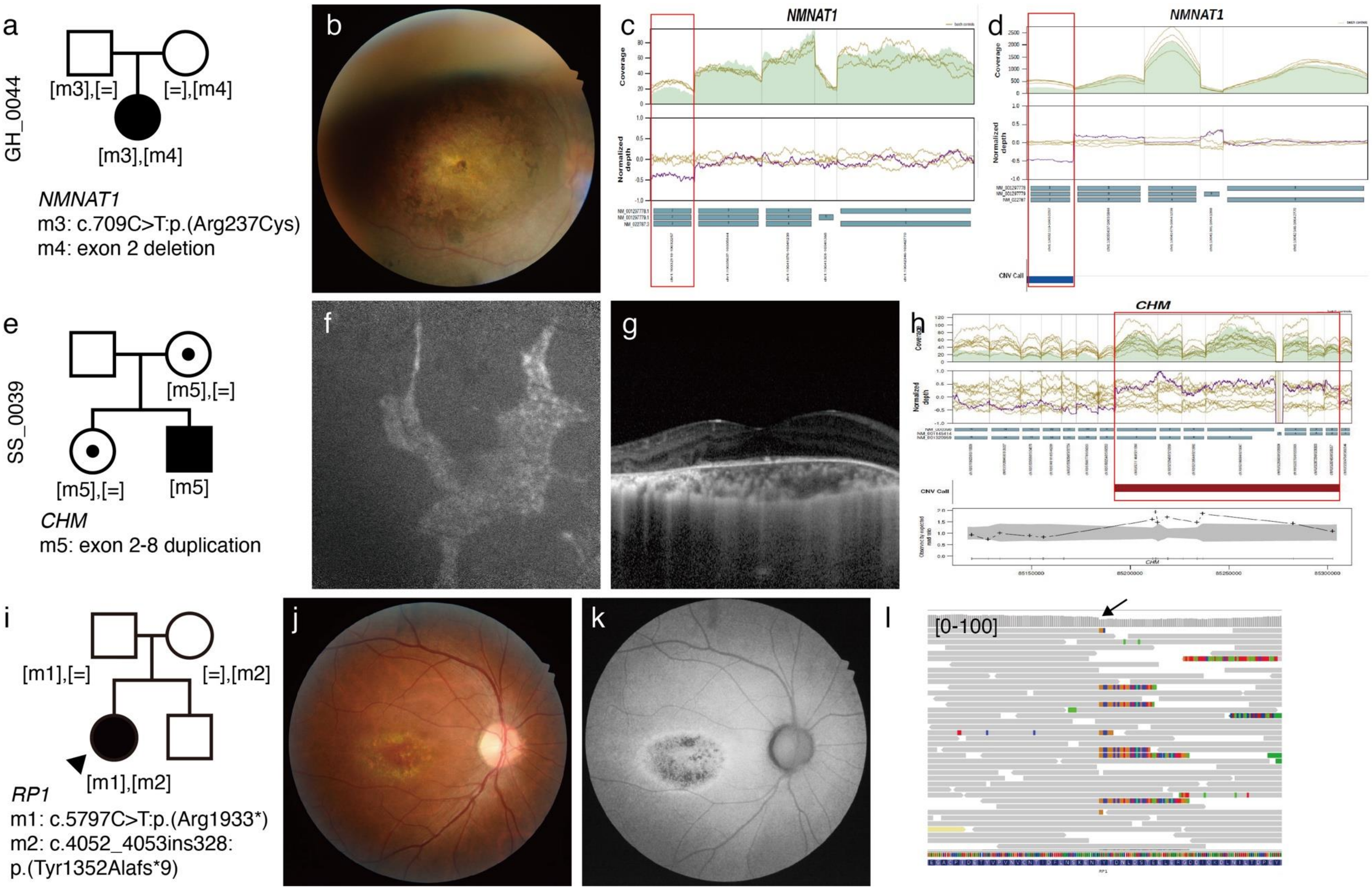

© 2024 Surl D et al. *JAMA Network Open.*

eFigure 10. Exome sequencing (ES) reanalysis identified hidden structural variants in unsolved patients with inherited retinal diseases. (a-d) A 1 year old girl with nystagmus and macular colobomatous degeneration came to our clinic. ES identified a single hit c.709C>T:p.(Arg237Cys) in *NMNAT1*. Because a second hit in *NMNAT1* is strongly suspected in this case, copy number variation re-analysis was done, and it revealed exon 2 deletion in *NMNAT1*. Confirmation of this single exon deletion was validated with high-depth targeted panel sequencing because no MLPA kit is available in *NMNAT1*. (e-h). The *CHM* exon 2-8 duplication was detected in initial ES analysis, but this duplication was not reported. Because the phenotype of this patient was consistent with choroideremia, ES reanalysis focused on *CHM* showed exon 2-8 duplication. This exon 2-8 duplication is out-of-frame, which results in frameshift. (i-l) ES reanalysis revealed *RP1*-Alu insertion in a patient with macular dystrophy. The mobile element insertion in this patient was successfully detected in both SCRAMble and *RP1*-Alu grep program.[6]

© 2024 Surl D et al. *JAMA Network Open.*

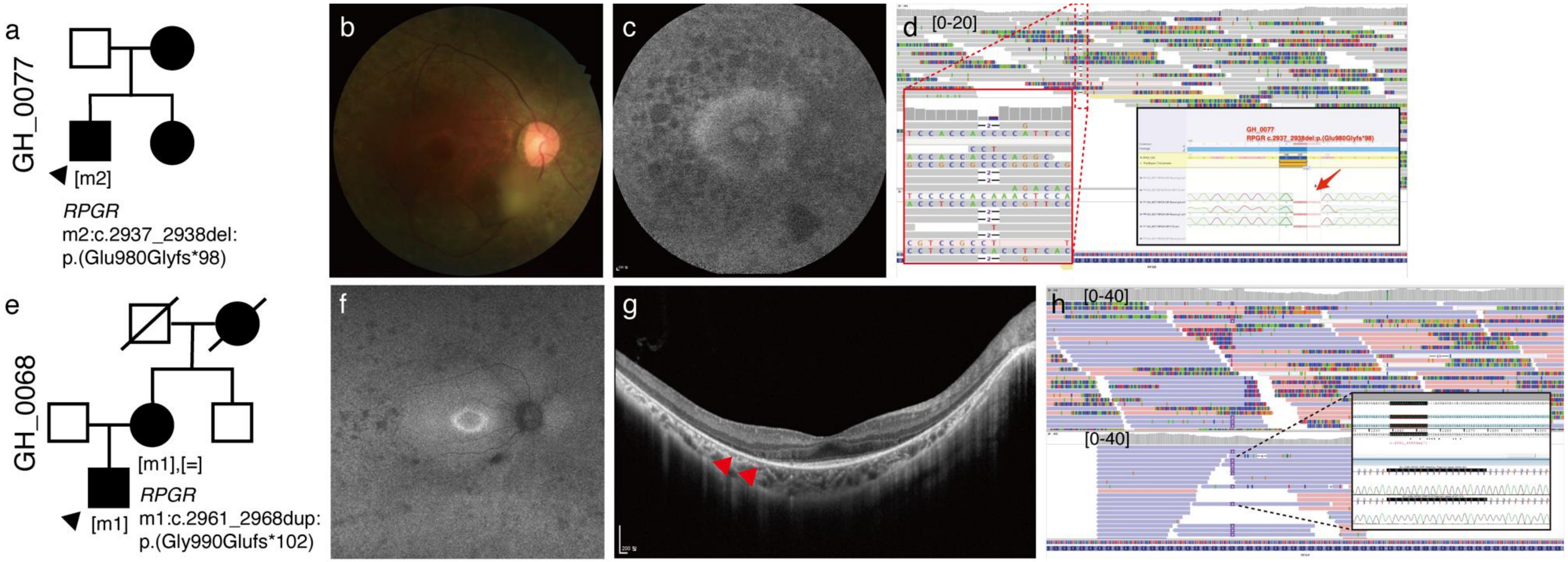

eFigure 11. Exome sequencing (ES) reanalysis and long-range PCR *RPGR* orf15 Sanger sequencing revealed pathogenic variants in repetitive difficult-to-map regions. (a) The pedigree revealed that it could be autosomal dominant, mitochondrial, or X-linked. No further family information was available. (b-c) Fundus photography and autofluorescence showed degeneration of retina mainly outside of macula. Ring hypo-fluorescence was also noted in fovea (right eye was shown). (d) This 2bp deletion was not called in both initial ES analysis and re-analysis. Only sanger sequencing of *RPGR* orf15 region identified c.2937_2938del:p.(Glu980Glyfs*98) variant. (e) Pedigree shows X-linked inheritance pattern and affected mother had unilateral retinitis pigmentosa, which is consistent with X-linked retinitis pigmentosa. (f) Autofluorescence (AF) image shows decreased density of AF with relatively preserved AF in fovea. (g) Optical coherence tomography shows loss of photoreceptors and attenuated outer retinal layers sparing fovea. (red arrowheads) (h) Initial analysis did not find any variant in this region, but ES reanalysis identified c.2961_2968dup:p.(Gly990Glufs*102) variant in *RPGR* orf15 region. This variant was called, but it was filtered owing to low depth. Integrative genomic viewer showed 8bp duplication in *RPGR* orf15 (Upper panel: mark duplicated bam file, Lower panel: Haplotypecaller bamout file).

© 2024 Surl D et al. *JAMA Network Open.*

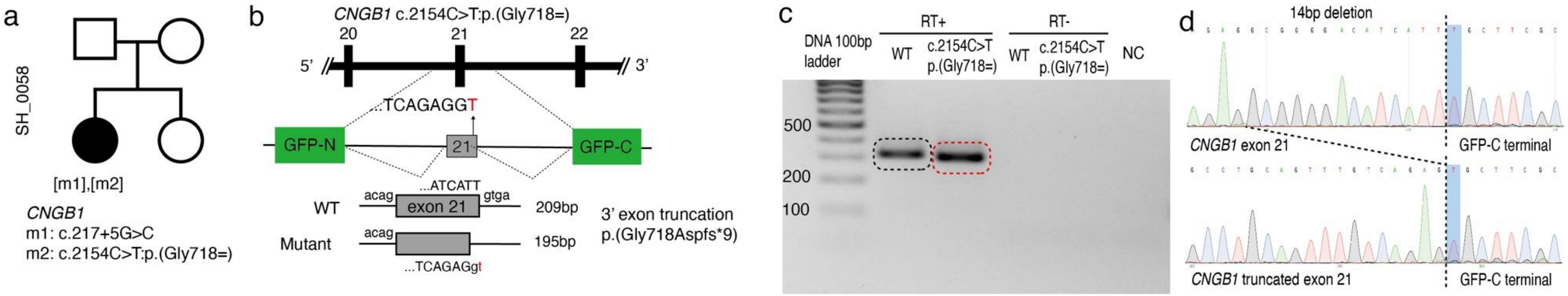

eFigure 12. Exome sequencing reanalysis with functional mini-gene splicing assay identified a 3' exon truncating synonymous variant. (a) The initial bioinformatic analysis identified a likely pathogenic c.217+5G>C variant and a synonymous c.2154C>T variant in *CNGB1*. The synonymous variant was previously reported in ClinVar, but it was classified as variant of uncertain significance. No other candidate variant was found in inherited retinal diseases genes (b) The 3' exon truncation (14 bp) was predicted in SpliceAI and Alamut splicing module analysis. (c-d) The minigene splicing assay showed that c.2154C>T:p.(Gly718=) variant causes 3' exon truncation, which results in frameshift truncation. The Sanger sequencing of gel products was matched to the predicted exon truncation.

© 2024 Surl D et al. *JAMA Network Open.*

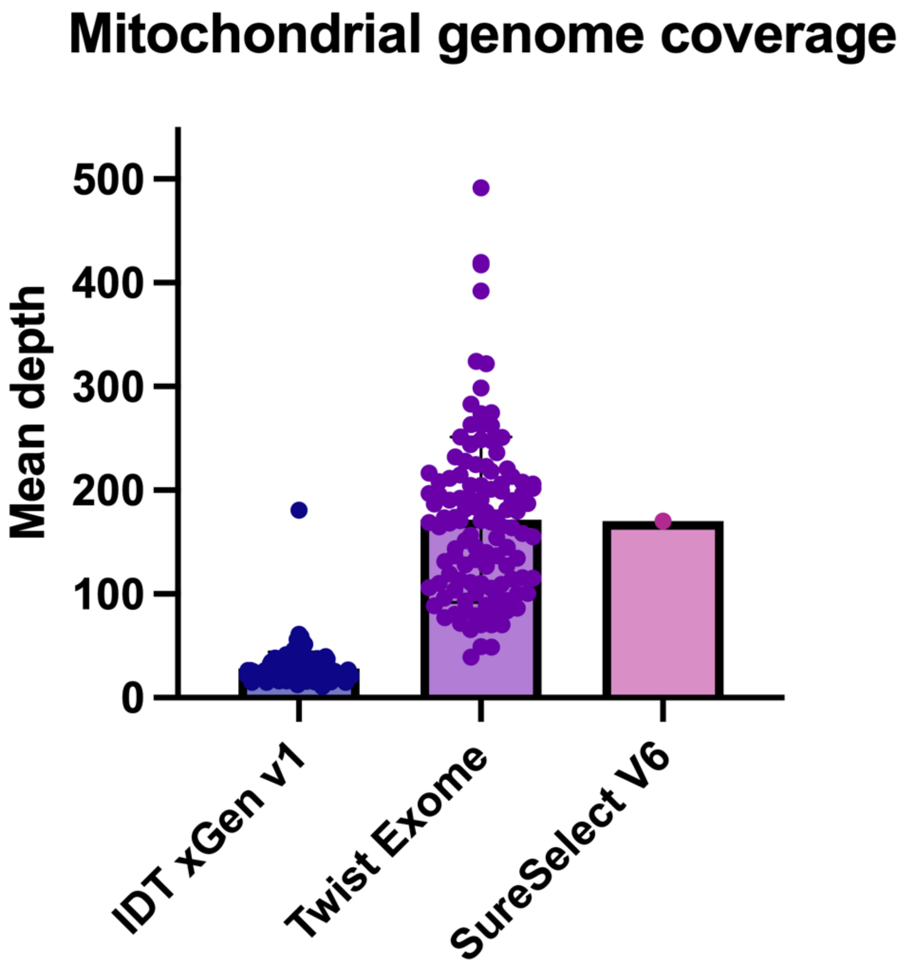

eFigure 13. Mitochondrial coverage according to each exome kit used in this study. Twist exome and SureSelect V6 exome kit showed generally better coverage compared to IDT xGen exome research v1 (P<0.001).

© 2024 Surl D et al. *JAMA Network Open.*

eReferences

1. Pais LS, Snow H, Weisburd B et al. seqr: A web-based analysis and collaboration tool for rare disease genomics. *Hum Mutat* 2022; 43(6):698-707.
2. Plagnol V, Curtis J, Epstein M et al. A robust model for read count data in exome sequencing experiments and implications for copy number variant calling. *Bioinformatics* 2012; 28(21):2747-2754.
3. Klambauer G, Schwarzbauer K, Mayr A et al. cn.MOPS: mixture of Poissons for discovering copy number variations in next-generation sequencing data with a low false discovery rate. *Nucleic Acids Res* 2012; 40(9):e69.
4. Babadi M, Fu JM, Lee SK et al. GATK-gCNV enables the discovery of rare copy number variants from exome sequencing data. *Nat Genet* 2023; 55(9):1589-1597.
5. Torene RI, Galens K, Liu S et al. Mobile element insertion detection in 89,874 clinical exomes. *Genet Med* 2020; 22(5):974-978.
6. Won D, Hwang JY, Shim Y et al. In Silico identification of a common mobile element insertion in exon 4 of RP1. *Sci Rep* 2021; 11(1):13381.
7. Scott HA, Place EM, Harper E et al. A high throughput splicing assay to investigate the effect of variants of unknown significance on exon inclusion. *medRxiv* 2022:2022.2011.2030.22282952.
8. Guo MH, Francioli LC, Stenton SL et al. Inferring compound heterozygosity from large-scale exome sequencing data. *Nat Genet* 2024; 56(1):152-161.
9. Jaganathan K, Kyriazopoulou Panagiotopoulou S, McRae JF et al. Predicting Splicing from Primary Sequence with Deep Learning. *Cell* 2019; 176(3):535-548.e524.
10. Fadaie Z, Whelan L, Ben-Yosef T et al. Whole genome sequencing and in vitro splice assays reveal genetic causes for inherited retinal diseases. *NPJ Genom Med* 2021; 6(1):97.
11. Olivier G, Corton M, Intartaglia D et al. Pathogenic variants in IMPG1 cause autosomal dominant and autosomal recessive retinitis pigmentosa. *J Med Genet* 2021; 58(8):570-578.

© 2024 Surl D et al. *JAMA Network Open.*
