# Supplementary material for: Clinician-Driven Reanalysis of Exome Sequencing Data From Patients With Inherited Retinal Diseases
Source: JAMA Netw Open. 2024 May 31;7(5):e2414198. doi: 10.1001/jamanetworkopen.2024.14198 (PMC11143468; doi:10.1001/jamanetworkopen.2024.14198)
Supplement: Supplement 2. — Data Sharing Statement [file jamanetwopen-e2414198-s002.pdf]

# Data Sharing Statement

Surl. Clinician-Driven Reanalysis of Exome Sequencing Data From Patients With Inherited Retinal Diseases. *JAMA Netw Open*. Published May 31, 2024.
doi:10.1001/jamanetworkopen.2024.14198

**Data**
**Data available:** Yes
**Data types:** Deidentified participant data
**How to access data:** jinuhan@yuhs.ac
**When available:** With publication

**Supporting Documents**
**Document types:** Statistical/analytic code
**How to access documents:** jinuhan@yuhs.ac
**When available:** With publication

**Additional Information**
**Who can access the data:** researchers whose proposed use of the data has been approved
**Types of analyses:** for a specified purpose
**Mechanisms of data availability:** with a signed data access agreement
